# Recapitulation and reversal of neuropsychiatric phenotypes in a mouse model of human endogenous retrovirus type W expression

**DOI:** 10.1038/s41380-025-02955-9

**Published:** 2025-03-18

**Authors:** Felisa Herrero, Celine Heeb, Michelle Meier, Han-Yu Lin, Flavia S. Mueller, Sina M. Schalbetter, Joel Gruchot, Ulrike Weber-Stadlbauer, Tina Notter, Hervé Perron, Patrick Küry, Urs Meyer

**Affiliations:** 1 Institute of Veterinary Pharmacology and Toxicology, University of Zurich, Zurich, Switzerland; 2 Department of Neurology, Medical Faculty, Heinrich-Heine-University Düsseldorf, Düsseldorf, Germany; 3 Institute of Pharmacology and Toxicology, University of Zurich, Zurich, Switzerland; 4 Neuroscience Center Zurich, University and ETH Zurich, Zurich, Switzerland; 5 GeNeuro, 18, chemin des Aulx, Plan-les-Ouates, 1228 Geneva, Switzerland; 6 Université de Lyon-UCBL, Lyon, France; 7 Department of Neurology, Inselspital, University Hospital and University of Bern, Bern, Switzerland

**Keywords:** Neuroscience, Schizophrenia, Molecular biology

## Abstract

Human endogenous retroviruses (HERVs) are inherited genetic elements derived from exogenous retroviral infections occurring throughout evolution. Accumulating evidence implicates increased expression of HERV type W envelope (HERV-W ENV) in psychiatric and neurodevelopmental disorders. To gain more mechanistic insights into the neurobiological disease pathways affected by HERV-W ENV expression, we took advantage of a mouse model that recapitulates the expression of the human-specific HERV-W ENV protein. Behavioral and cognitive phenotyping of transgenic (TG) mice expressing HERV-W ENV and wild-type (WT) controls showed that expression of this retroviral envelope caused deficits in numerous functional domains, including repetitive behavior, social and object recognition memory, and sensorimotor gating. Genome-wide RNA sequencing of hippocampal tissue demonstrated that transgenic expression of HERV-W ENV led to transcriptomic alterations that are highly relevant for psychiatric and neurodevelopmental disorders, cognitive functions, and synaptic development. Differential gene expression in TG mice encompassed a downregulation of several genes associated with schizophrenia and autism spectrum disorder, including *Setd1a*, *Cacna1g*, *Ank3*, and *Shank3*, as well as a downregulation of histone methyltransferase genes that belong to the Set1-like histone H3 lysine 4 (H3K4) methyltransferase family (*Kmt2a*, *Kmt2b* and *Kmt2d*). Concomitant to the latter, HERV-W ENV mice displayed increased enzymatic activity of lysine-specific demethylase-1 (LSD1), increased H3K4 mono-methylation, and decreased H3K4 di- and tri-methylation in the hippocampus. Importantly, pharmacological inhibition of LSD1 through oral ORY-1001 treatment normalized abnormal H3K4 methylation and rescued the behavioral and cognitive deficits in HERV-W ENV mice. In conclusion, our study suggests that the expression of HERV-W ENV has the capacity to disrupt various behavioral and cognitive functions and to alter the brain transcriptome in a manner that is highly relevant to neurodevelopmental and psychiatric disorders. Moreover, our study identified epigenetic pathways that may offer avenues for pharmacological interventions against behavioral and cognitive deficits induced by increased HERW-W expression.

## Introduction

Human endogenous retroviruses (HERVs) are inherited retroviral genomic elements that integrated into the human genome through germline infections and insertions during evolution [1]. They belong to retrotransposons and use a “copy-and-paste” mechanism to integrate their DNA product into the genome based on reverse transcription of an RNA intermediate [2]. It is estimated that HERVs comprise 5–8% of the human genome, which readily surpasses the 1–2% of human DNA-containing protein-coding sequences [3]. Under physiological conditions, the majority of HERVs are thought to be in a dormant state and suppressed via epigenetic silencing, involving DNA methylation, histone modifications and non-coding RNAs [4]. Yet, under certain pathological conditions, some HERVs can be activated and contribute to disorders of the central nervous system (CNS) [5, 6]. As reviewed in detail elsewhere [5, 6], activation of HERVs may include prior exposure to certain infections, such as Epstein-Barr virus (EBV), *Toxoplasma gondii*, and SARS-CoV2. Furthermore, HERVs can be activated by environmental stimuli, including stress [7, 8], environmental toxins [9, 10], and inflammatory challenges [6, 11]. Multiple mechanisms for HERV activation and expression exist, including epigenetic de-repression of HERV promoter regions, binding of stress- and immune-responsive transcription factors to response elements in HERV promoters, chromatin remodeling, and cis-activation of HERV loci through genomic integration of exogenous retroviruses [4, 12].

Among HERVs, the HERV type-W (HERV-W) family is one of the most intensively investigated, especially with regards to its pathophysiological roles in neurological disorders, such as multiple sclerosis (MS) [5, 6, 13, 14]. HERV-W was originally termed “MS-associated retrovirus (MSRV)” after its initial discovery in leptomeningeal cells of MS patients [15–17]. Integration of HERV-W into the human genome resulted from retroviral infection of ancestorial germ line cells over 20 millions years ago [18]. Whereas many HERVs do not contain open reading frames (ORFs) for complete retroviral proteins, HERV-W retains coding sequences for functional HERV-W proteins [5, 19]. In addition, they have recently been shown to regulate the transcription of regulatory non-coding RNAs, further adding to their biological activity in health and disease [20, 21]. The pathogenic activity of HERV-W has been mainly related to its envelope (ENV) protein, which can affect CNS structures and functions through multiple mechanisms [5, 6]. Besides others, these include effects on glial cell differentiation and polarization [14], myelin repair [22], neurotransmitter metabolism [23], and synaptic functionality [24].

Accumulating evidence implicates increased expression of HERV-W in neurodevelopmental and psychiatric disorders, including schizophrenia, bipolar disorder and autism spectrum disorder (ASD) [25–34]. Moreover, persistent HERV-W expression has also been associated with a functional deterioration in the long-Covid syndrome [35, 36]. Thus far, however, the available evidence implicating HERV-W in these disorders is mostly based on correlative studies assessing HERV-W ENV antigenemia and/or gene expression in patients relative to controls or in relation to disease progression [25–36]. Hence, mechanistic insights into the neurobiological disease pathways affected by HERV-W is still missing in this context.

To seek causal evidence for a role of HERV-W in disrupting brain and behavioral functions pertaining to neurodevelopmental and psychiatric disorders, we took advantage of a mouse model that recapitulates the expression of the human-specific HERV-W ENV protein. In this model, transgenic mice express the MSRV-pV14 ENV sequence, which features the HERV-W ENV ORF  and the 3’ long terminal repeat (3’LTR) under the CAG promoter, allowing ubiquitous expression of the HERV-W ENV protein [14, 37]. This transgenic mouse line is currently the only existing mouse model of human-specific HERV-W ENV expression and allows mechanistic insights into disease pathways affected by HERV-W [14, 37].

Using this model, we performed extensive behavioral and cognitive phenotyping involving tests that are relevant to neurodevelopmental and psychiatric disorders, including tests for locomotor activity, innate anxiety-like behavior, social approach behavior and social recognition memory, sensorimotor gating, and novel object recognition. We also conducted next-generation RNA sequencing and histone modification analyses to identify transcriptomic and epigenetic alterations in mice expressing HERV-W ENV. Finally, we implemented a pharmacological approach with the aim to correct the behavioral and cognitive deficits emerging in mice expressing the HERV-W ENV protein. Collectively, our study shows that the expression of this retroviral element has the capacity to disrupt various behavioral and cognitive functions and to alter the brain transcriptome in a manner that is highly relevant to neurodevelopmental and psychiatric disorders. Moreover, our study identifies epigenetic pathways that may offer avenues for pharmacological interventions against behavioral and cognitive deficits induced by increased HERW-W expression.

## Methods

### Animals

Transgenic C57BL6/J;129P2/Ola-Hprt mice (referred to as CAG^HERV-Wenv^ TG mice) and wild-type (WT) littermates were used throughout this study. The generation of this mouse line has been fully described elsewhere [14, 37]. In TG mice, the MSRV-pV14-env transgene (GenBank, AF331500-1) is expressed under the control of the ubiquitous CAG promoter and is inserted in the hypoxanthine phosphoribosyltransferase (HPRT) locus of the murine X-chromosome [14, 37]. Male hemizygous TG mice and WT littermates were generated by breeding WT males with heterozygous TG females. Since this breeding strategy did not yield homozygous female TG mice as a result of the X-chromosome insertion, the present study was conducted using male hemizygous TG mice and male WT littermates only.

Animal genotypes were determined using genomic polymerase chain reaction (PCR), as described before [14, 37]. Expression of the HERV-W ENV transcripts in TG mice was confirmed by quantitative real-time PCR analyses (qRT-PCR) (Supplementary Fig. S1) using methods described in the Supplementary Information. All animals were kept in a temperature- and humidity-controlled (21 ± 1 °C, 55 ± 5%), specific pathogen free (SPF) holding facility under a reversed light-dark cycle (lights off: 09.00–21.00) and were maintained under *ad libitum* standard rodent chow and water. The animals were group-housed (3–5 animals per cage) in individually ventilated cages (IVCs), as described before [38]. All procedures described in the present study had been previously approved by the Cantonal Veterinarian’s Office of Zurich, and all efforts were made to minimize the number of animals used and their suffering.

### Behavioral and cognitive testing

In a first cohort of WT and TG mice (Supplementary Table 1), behavioral and cognitive testing commenced when they reached 12 weeks of age and included tests for locomotor activity and innate anxiety-like behavior (open field test and light-dark box test), repetitive behavior (marble burying test), sociability and social memory (social interaction test), declarative memory for objects (novel object recognition test), and sensorimotor gating (prepulse inhibition [PPI] of the acoustic startle reflex). Each animal was tested repeatedly using the same order of testing (1. open field test; 2. light-dark box test; 3. marble burying test; 4. social interaction test; 5. novel object recognition test; 6. PPI test), with a resting phase of 2–3 days between individual tests. Using a second cohort of WT and TG mice (Supplementary Table 1), we further assessed sociability and social memory, novel object recognition memory, and PPI of the acoustic startle reflex in WT and TG mice during adolescence, i.e., when they were 4 to 6 week of age. Again, each animal was tested repeatedly using the same order of testing (1. social interaction test; 2. novel object recognition test; 3. PPI test), with a resting phase of 3 days between individual tests. A detailed description of the methodological procedures and rationale of inclusion are provided for each behavioral test in the Supplementary Information.

### ORY-1001 treatment

A third cohort of WT and TG mice (Supplementary Table 1) was used to assess the therapeutic effects of ORY-1001, which acts as a selective inhibitor of the epigenetic enzyme lysine-specific demethylase-1 (LSD1) [39]. ORY-1001 (Cayman chemicals, Michigan, USA) or vehicle (VEH) was administered *per os* using the micropipette-guided drug administration (MDA) method (Supplementary Information), a stress-free alternative to oral gavage for chronic *per os* treatments in mice [40]. When the animals reached 10 weeks of age, ORY-1001 was given daily at 0.01 mg/kg for two weeks prior to the commencement of behavioral and cognitive testing, after which it was maintained throughout the testing period. During the behavioral testing phase, the drug and the corresponding vehicle were always given after testing, that is, at the end of the reversed light-dark cycle between 19:00 and 21:00. The dose of ORY-1001 was selected based on previous studies in mice, showing that it effectively rescues ASD- and schizophrenia-related behavioral and cognitive deficits without producing detectable side effects in control animals at this dose [39, 41, 42].

### Transcriptomic analysis

We performed next-generation RNA sequencing (RNAseq) to compare genome-wide transcriptional changes in the prefrontal cortex (PFC) and hippocampus of adult TG relative to WT mice (Supplementary Table 1). These brain regions were selected based on the results obtained in the preceding behavioral and cognitive phenotyping and were extracted from behaviorally naïve mice at the age of 12 weeks. Following RNA extraction quality control, sequencing was performed using NovaSeq6000 (llumina, Inc, California, USA) as described in the Supplementary Information. Differentially expressed genes (DEGs) were identified using the R package edgeR from Bioconductor Version 3.10 with false discovery rate (FDR)-corrected p-values set to a 10% threshold (*q* < 0.1) and *p* < 0.0012 (Supplementary Information). Functional network prediction was generated using Ingenuity Pathway Analysis (QIAGEN, Redwood City, CA, USA) [43, 44], as described in the Supplementary Information. Differential gene expression of selected genes was validated through qRT-PCR using the Biorad CFX384^TM^ Real-Time System (Biorad, Hercules, California, USA) (Supplementary Information). Probe and primer sequences of each gene of interest are summarized in Supplementary Table 2.

### Analyses of histone H3 lysine 4 (H3K4) methylation

As described in the Supplementary Information, standard Western blot techniques were used to assess mono-, di-, and tri-methylation of H3K4 (H3K4me1, H3K4me2, and H3K4me3) and LSD1 in the hippocampus of behaviorally naïve WT and TG mice (Supplementary Table 1). The blots were developed using a ChemiDoc XRS+ System (Bio-Rad, catalogue number: 1708299) and were analyzed with reference to H3 as the housekeeping control. To confirm altered H3K4 methylation using another technique, we performed immunohistochemistry of H3K4me2 in the hippocampus of WT and TG mice. Since the Western blot and immunohistochemical analyses of H3K4me2 yielded highly consistent results, we restricted the immunohistochemical validation of altered H3K4 methylation to the analysis of H3K4me2. Methodological details regarding tissue preparation, immunofluorescence staining and microscopy are provided in the Supplementary Information. Antibodies used for Western blot analyses are summarized in Supplementary Table 3.

### Estimation of synaptic density via co-localization analysis

We estimated the density of excitatory and inhibitory synapses in the hippocampus of adult WT and TG mice via co-localization analysis. To this end, we used the presynaptic marker vesicular glutamate transporter 1 (VGLUT1) and the postsynaptic marker postsynaptic density protein 95 (PSD-95) for excitatory neurons, whereas vesicular γ-aminobutyric acid transporter (VGAT) and Gephyrin were used as inhibitory presynaptic and postsynaptic markers, respectively. Methodological details regarding tissue preparation, immunofluorescence staining and microscopy are provided in the Supplementary Information.

### Enzymatic activity of LSD1

The enzymatic activity of LSD1 was measured in the hippocampus of behaviorally naïve WT and TG mice using the Epigenase™ LSD1 demethylase activity/inhibition assay (EpigenTek; Farmingdale, NY, USA), as described in the Supplementary Information.

### Statistical analysis

All statistical analyses of behavioral, cognitive, Western blot, RT-qPCR, and immunohistochemical data were performed using Statistical Package for the Social Sciences (SPSS) Statistics (version 29.0, IBM, Armonk, NY, USA) and Prism (version 10.0; GraphPad Software, La Jolla, California), with statistical significance set at *p* < 0.05 unless specified otherwise. All data met the assumptions of normal distribution and equality of variance. Exclusion of animals was not applied. All data involving two independent variables were analyzed using independent Student’s *t* tests (two-tailed), whereas data encompassing more than two independent variables were analyzed using analysis of variance (ANOVA), followed by Tukey’s or Šidák post-hoc tests for multiple comparisons whenever appropriate. Transcriptomic data were analyzed as described above, using FDR correction set at a 10% threshold (*q* < 0.1). A detailed description of the statistical analyses is provided in the Supplementary Information.

## Results

### Transgenic expression of HERV-W ENV leads to adult behavioral and cognitive deficits

**Fig. 1:**
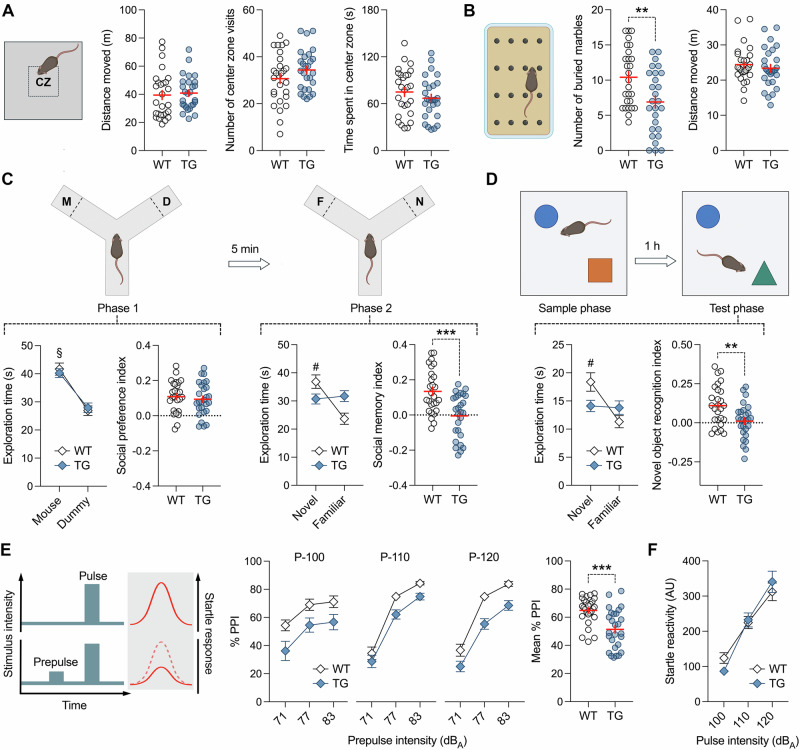
Behavioral and cognitive phenotypes of adult mice expressing HERV-W ENV. All data were obtained from adult male transgenic CAG^HERV-Wenv^ mice (TG) and wild-type (WT) littermates. **A** Total distance moved, distance moved in the center zone (CZ) and time spent in the CZ during the open field test. **B** Number of buried marbles and distance moved during the marble burying test. **p < 0.01, based on independent t-test (*t*_(52)_ = 2.97, 2-tailed). **C** Phase 1 (D = dummy object, M = unfamiliar mouse) and phase 2 (F = familiar mouse; N = novel mouse) of the social interaction test, which assessed sociability and social memory, respectively. The line plots show the time spent with the unfamiliar mouse or dummy object in phase 1 of the test, or with the novel or familiar mouse in phase 2 of the test. The scatter plots depict the social preference index (values > 0 represent a preference toward the unfamiliar mouse) in phase 1 and the social memory index (values > 0 represent a preference toward the novel mouse) in phase 2 of the test. ^§^*p* < 0.001, reflecting the significant main effect of object (*F*_(1,52)_ = 57.13) in repeated-measures ANOVA; ^#^*p* < 0.001, reflecting the significant difference between novel and familiar mouse exploration in WT mice, based on Šidák post-hoc test after the presence of a significant 2-way interaction between genotype and object (*F*_(1,52)_ = 13.68, *p* < 0.001) in repeated-measures ANOVA; ***p < 0.001, based on independent t-test (*t*_(52)_ = 4.09, 2-tailed). **D** Object recognition memory in the novel object recognition test, in which the animals were required to discriminate a novel object from a previously familiarized object. The line plot shows the time spent with the novel or familiar object during the test phase, whereas the scatter plot depicts the novel object recognition index (values > 0 represent a preference toward the novel object). ^#^*p* < 0.001, reflecting the significant difference between novel and familiar object exploration in WT mice, based on Šidák multiple post-hoc test following a significant 2-way interaction between genotype and object (*F*_(1,52)_ = 10.52, *p* < 0.001) in repeated-measures ANOVA; **p < 0.001, based on independent t-test (*t*_(52)_ = 3.06; 2-tailed). **E** Prepulse inhibition (PPI) test of sensorimotor gating using 3 prepulse intensities (71, 77 and 83 dB_A_) and 3 pulse intensities (P-100, P-110 and P-120, which correspond to 100, 110 and 120 dB_A_). The line plots show % PPI as a function of prepulse and pulse intensities, whereas the scatter plot depicts the mean % PPI across all prepulse and pulse intensities. ***p < 0.001, reflecting the significant main effect of genotype (*F*_(1,52)_ = 19.55) in repeated-measures ANOVA. **F** Acoustic startle reactivity (in arbitrary units, AU) to 100-, 110- and 120-dB_A_ pulse stimuli. All scatter plots show individual mice with overlaid group means ± s.e.m; all line plots show group means ± s.e.m. All data are based on *n* = 27 mice in each genotype per test.

We used a behavioral test battery to investigate whether HERV-W ENV expression in mice alters adult behavioral and cognitive functions relevant to psychiatric disorders. First, we assessed basal locomotor activity and innate anxiety-like behavior using the open field test [45]. In this test, TG mice did not differ from WT controls in terms of the total distance moved, number of center zone visits, or time spent in the center zone (Fig. 1A), demonstrating that transgenic expression of HERV-W ENV does not affect locomotor activity or innate anxiety-like behavior in a novel environment. Consistent with these findings, there were no differences between WT and TG mice in terms of the total distance moved, distance moved in the light compartment, and time spent in the light compartment during the light-dark box test (Supplementary Fig. S2), which is another test used to asses innate anxiety-like behavior [45]. To assess repetitive, compulsive-like behaviors, we used the marble burying test [46, 47], which is commonly used in preclinical research of ASD and other neurodevelopmental disorders [48, 49]. Although the number of buried marbles was found to be variable in both genotypes, adult TG mice buried significantly less marbles than WT controls (Fig. 1B). This effect emerged in the absence of changes in locomotor activity during the marble burying test (Fig. 1B), suggesting that reduced marble burying in adult TG mice represented a genuine deficit in repetitive, compulsive-like behavior [46, 47].

We then used a two-phase social interaction test to assess sociability and social memory in adult TG and WT mice. Sociability and/or social memory are disrupted in several psychiatric and neurodevelopmental disorders, including schizophrenia and ASD [50, 51], as well as in rodent models of these disoders [52, 53]. In the first phase of the social interaction test, where the animals were allowed to freely explore a nonfamiliar mouse or an inanimate dummy object, TG and WT mice both displayed a clear preference toward the nonfamiliar mouse, indicating intact sociability in either genotype (Fig. 1C). When allowed to explore a novel versus familiar mouse in the second phase of the test, only WT mice showed a preference toward the novel mouse (Fig. 1C). In contrast, TG mice were unable to discriminate between the novel and familiar mouse (Fig. 1C), demonstrating impaired social recognition memory in adult mice expressing HERV-W ENV.

Consistent with the deficit in social recognition memory, adult TG mice also displayed an impairment in novel object recognition memory. In the novel object recognition test, which is commonly used to identify transdiagnostic deficits in declarative memory [54], TG and WT mice were first allowed to freely explore two distinct objects, which differed in terms of shape and color (sample phase). In the subsequent test phase, when one of the familiarized objects was replaced by a novel object, only WT mice showed a clear preference toward the novel object, whereas TG mice failed to discriminate between the two objects (Fig. 1D). These data show that transgenic expression of HERV-W ENV in mice disrupts recognition memory for objects at adult age.

In a last step, we examined the effects of HERV-W ENV expression on PPI of the acoustic startle reflex, a form of sensorimotor gating known to be impaired in schizophrenia and other psychiatric disorders [55, 56]. As shown in Fig. 1E, adult TG mice displayed a significant reduction in PPI as compared to WT controls. This deficit was noticeable across all pulse levels, leading to a deficit in mean % PPI (Fig. 1E). The reduction in PPI was not associated with differences in acoustic startle reactivity per se (Fig. 1E), demonstrating that transgenic expression of HERV-W ENV leads to an adult deficit in sensorimotor gating in the absence of concomitant changes in startle reactivity.

Taken together, our results demonstrate that mice expressing HERV-W ENV display a range of behavioral and cognitive abnormalities at adult age. To explore the ontogeny of these abnormalities, we assessed sociability and social memory, novel object recognition memory, and PPI of the acoustic startle reflex in WT and TG mice during adolescence, i.e., when they were 4 to 6 week of age. As summarized in Supplementary Fig. S3, we found that adolescent TG mice expressing HERV-W ENV did not significantly differ from age-matched WT controls in any of these behavioral and cognitive measures. Thus, deficits in social memory, novel object recognition memory, and PPI of the acoustic startle reflex appear to emerge only in adult mice expressing HERV-W ENV, suggesting that the expression of this retroviral entity alters the developmental trajectories of behavioral and cognitive functions in a manner particularly relevant to psychiatric disorders with onset in early adulthood.

### Transcriptomic alterations in mice expressing HERV-W ENV

To identify molecular abnormalities in mice expressing HERV-W ENV, we performed genome-wide RNAseq using PFC and hippocampal samples from adult TG and WT mice. The PFC and hippocampus were selected based on their involvement in social memory, recognition memory for objects, and sensorimotor gating [57–61], all of which were affected by transgenic expression of HERV-W ENV (Fig. 1). In keeping with the adult onset of behavioral and cognitive changes in mice expressing HERV-W ENV (Fig. 1, Supplementary Fig. S3), genome-wide RNAseq was conducted using brain samples from adult WT and TG mice.

**Fig. 2:**
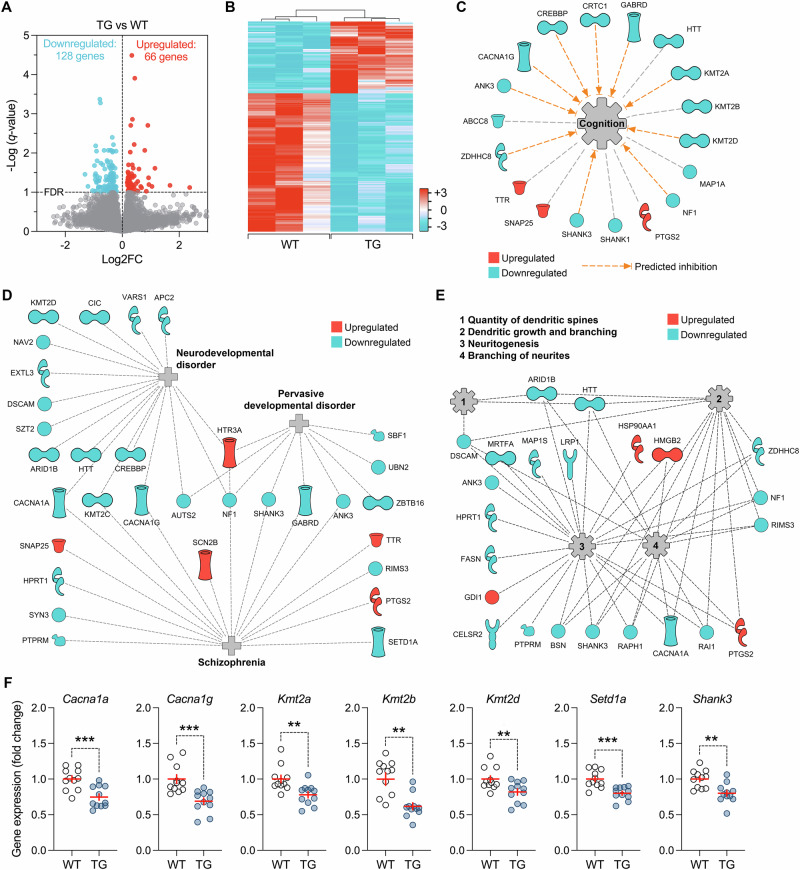
Transcriptomic alterations in the hippocampus of adult mice expressing HERV-W ENV. Next-generation RNA sequencing was used to identify genome-wide transcriptional changes in the bulk hippocampus of adult male transgenic CAG^HERV-Wenv^ mice (TG) and wild-type (WT) littermates. **A** The volcano plot depicts the statistical significance (-log [q-value]) *versus* the magnitude of gene expression changes (log2 fold change, Log2FC) in TG relative to WT mice (*n* = 3 per genotype). Using an FDR threshold of *q* < 0.1 and *p* < 0.0012, 128 genes (blue dots) and 66 genes (red dots) were down- and upregulated, respectively, in in TG relative to WT mice. **B** Hierarchical clustering of differentially expressed genes in TG relative to WT mice. The color-coded key denotes downregulation (blue) and upregulation (red) in terms of log2 ratios. **C** Graphical representation of differentially expressed genes annotated with the functional module “cognition” in Ingenuity Pathway Analysis. Predicted inhibition resulting from gene deregulation in TG mice is represented by dashed, orange arrows. **D** Graphical representation of differentially expressed genes annotated with the disease modules “neurodevelopmental disorder”, “pervasive developmental disorder”, and “schizophrenia”, as revealed by Ingenuity Pathway Analysis. **E** Graphical representation of differentially expressed genes annotated with the functional modules “quantity of dendritic spines”, dendritic growth and branching”, “neuritogenesis”, and “branching of neurites”, as revealed by Ingenuity Pathway Analysis. **F** Confirmation of gene expression changes in bulk hippocampal samples from TG relative to WT mice (*n* = 11 male mice per ge*n*otype) using qRT-PCR analyses. The scatter plots show fold changes of selected genes that were found to be downregulated in preceding RNA sequencing. **p < 0.01 and ***p < 0.001, based on independent *t*-tests (*Cacna1a*: *t*_(20)_ = 3.94; *Cacna1g*: *t*_(20)_ = 4.01; *Kmt2a*: *t*_(20)_ = 3.19; *Kmt2b*: *t*_(20)_ = 3.77; *Kmt2d*: *t*_(20)_ = 2.81; *Setd1a*: *t*_(20)_ = 4.34; *Shank3*: *t*_(20)_ = 3.49).

Using a FDR threshold of *q* < 0.1 and *p* < 0.0012, only five genes (*Cd99l2, Fxyd2, Rnf26rt, Scn2b*, and *Zbtb16*) were differentially expressed in the PFC of TG mice relative to WT controls (Supplementary Table 4), indicating that HERV-W ENV expression excerts only a limited impact on prefrontal gene expression at adult age. In the bulk hippocampus, however, we found that 128 and 66 genes were down- and upregulated, respectively, in TG mice relative to WT controls (Fig. 2A**;** Supplementary Table 5). These DEGs clustered according to genotype (Fig. 2B) and included a downregulation of several histone methyltransferase genes, including members of the Set1-like H3K4 methyltransferase family (*Kmt2a*, *Kmt2b, Kmt2c*, and *Kmt2d*) and SET-domain-containing 1 A (*Setd1a*). The latter is a chromatin remodeler that influences gene expression through the modulation of H3K4me2 and H3K4me3 states and is strongly implicated in schizophrenia [62–64] and other neurodevelopmental disorders [65, 66]. Intriguingly, transgenic expression of HERV-W ENV also led to impaired expression of several other genes that are implicated as genetic risk factors of schizophrenia and ASD, including *Cacna1g* [64, 67], *Ank3* [68], *Shank1* [69, 70], and *Shank3* [69, 70] (Fig. 2; Supplementary Table 5). We confirmed the downregulation of these genes in the bulk hippocampus of TG mice relative to WT controls using qRT-PCR analyses conducted in an independent cohort of animals (Fig. 2F). Furthermore, additional qRT-PCR analyses of key candidate genes were conducted separately for dorsal and ventral hippocampal samples (Supplementary Fig. S4). These analyses revealed a consistent pattern of gene deregulation along the dorsoventral axis of the hippocampus in TG mice compared to WT controls (Supplementary Fig. S4). Indeed, with the notable exception of *Cacna1g* and *Setd1a*, which were only downregulated in the ventral and dorsal hippocampus, respectively, mRNA expression of all other genes was consistently reduced in dorsal and ventral hippocampus of TG mice compared to WT controls (Supplementary Fig. S4).

We used Ingenuity Pathway Analysis (IPA) to identify functional networks that are altered in the hippocampus of TG mice relative to WT controls. Providing a molecular substrate for the cognitive deficits in TG mice (Fig. 1), IPA revealed gene sets that were annotated with the inhibition of cognition (Fig. 2C). Moreover, the identified DEGs were annotated with the IPA disease modules “neurodevelopmental disorder”, “pervasive developmental disorder”, and “schizophrenia” providing molecular links between transgenic expression of HERV-W ENV and these disorders. In addition to including members of the Set1-like H3K4 methyltransferase family (*Kmt2a*, *Kmt2b, Kmt2c*, and *Kmt2d*), these modules also encompassed rare variant genes associated with schizophrenia and ASD (*Setd1a*, *Cacna1g*, *Ank3*, *Shank1*, and *Shank3;* Fig. 2C). Finally, IPA revealed DEGs that were annotated with the functional modules “quantity of dendritic spines”, dendritic growth and branching”, “neuritogenesis”, and “branching of neurites” (Fig. 2D). Together, these findings show that transgenic HERV-W ENV expression in mice leads to transcriptomic alterations pertaining to psychiatric and neurodevelopmental disorders, cognitive functions, and synaptic development.

### Transgenic expression of HERV-W ENV reduces the synaptic density of excitatory neurons in the hippocampus

**Fig. 3:**
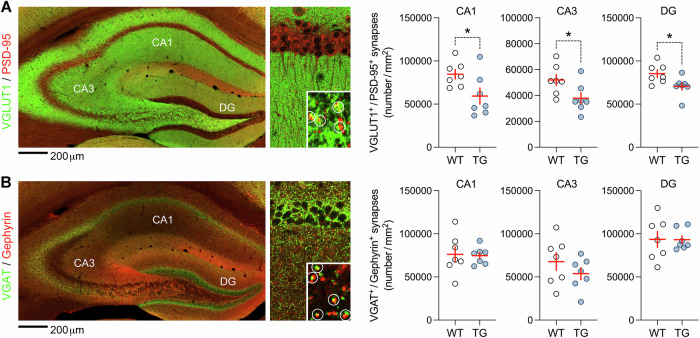
Synaptic alterations in adult mice expressing HERV-W ENV. Synaptic densities were quantified the CA1, CA3, and DG subregions of the hippocampus from adult male transgenic CAG^HERV-Wenv^ mice (TG) and wild-type (WT) littermates. **A** The photomicrograph shows a representative double-immunofluorescence stain using VGLUT1 (green) as presynaptic and PSD-95 (red) as postsynaptic markers of excitatory neurons. Examples of VGLUT1^+^/PSD-95^+^ co-localizing synapses are highlighted by white circles in magnified sections. The scatter plots show the density (number/mm^2^) of VGLUT1^+^/PSD-95^+^ excitatory synapses in each subregion of the hippocampus from WT and TG mice. *p < 0.05 (CA1: *t*_(12)_ = 2.35; CA3: *t*_(12)_ = 2.32; DG: *t*_(12)_ = 2.43), based on independent *t*-tests (two-tailed). **B** The photomicrograph shows a representative double-immunofluorescence stain using VGAT (green) as presynaptic and Gephyrin (red) as postsynaptic markers of inhibitory neurons. Examples of VGAT ^+^/Gephyrin^+^ co-localizing synapses are highlighted by white circles in magnified sections. The scatter plots show the density (number/mm^2^) of VGAT ^+^/Gephyrin^+^ inhibitory synapses in each subregion of the hippocampus from WT and TG mice. All scatter plots show individual mice with overlaid group means ± s.e.m.; *n* = 7 per ge*n*otype.

We conducted immunohistochemical analyses of synaptic densities to validate some of the transcriptomic changes that were indicative of synaptic deficits in adult mice expressing HERV-W ENV. Using co-localization analyses of presynaptic and postsynaptic markers, we estimated the density of excitatory and inhibitory synapses in the hippocampus of adult WT and TG mice. To this end, we used the presynaptic marker VGLUT1 and the postsynaptic marker PSD-95 for excitatory neurons, whereas VGAT and Gephyrin were used as inhibitory presynaptic and postsynaptic markers, respectively. As shown in Fig. 3A, we found that adult mice expressing HERV-W ENV displayed decreased densities of VGLUT1^+^/PSD-95^+^ excitatory synapses in the cornu Ammonis (CA)1, CA3, and dentate gyrus (DG) subregions of the hippocampus. On the other hand, the densities of VGAT^+^/Gephyrin^+^ inhibitory synapses did not differ between WT and TG mice (Fig. 3B). These data provide additional support for the hypothesis that HERV-W ENV expression affects synaptic structures in the hippocampus and suggest that excitatory synapses are particularly vulnerable to this endogenous retroviral element.

### Transgenic expression of HERV-W ENV leads to abnormal H3K4 methylation

**Fig. 4:**
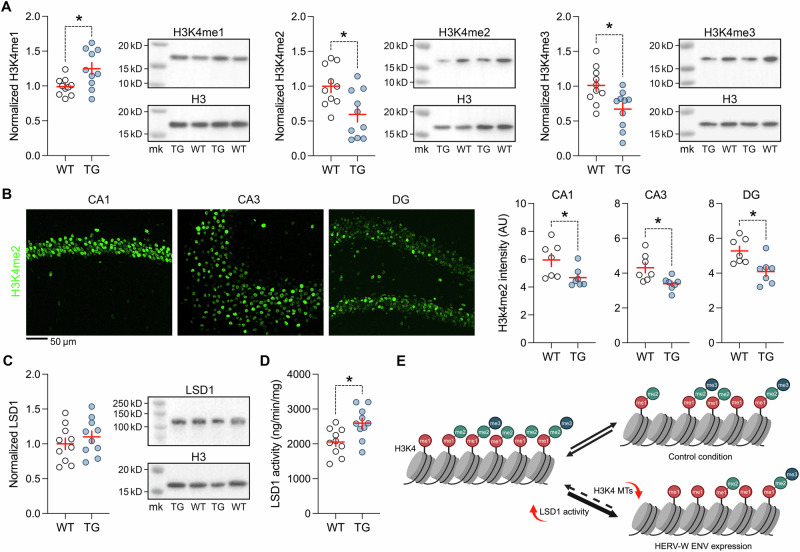
Abnormal H3K4 methylation and LSD1 activity in adult mice expressing HERV-W ENV. All data were generated using hippocampal samples of adult male transgenic CAG^HERV-Wenv^ mice (TG) and wild-type (WT) littermates. **A** Western blot analysis of H3K4me1 (*p < 0.05, *t*_(18)_ = 2.25, 2-tailed), H3K4me2 (*p < 0.01, *t*_(18)_ = 2.97, 2-tailed) and H3K4me3 (*p < 0.05, *t*_(18)_ = 2.58, 2-tailed), normalized to H3 housekeeping control. The photographs show representative Western blots using H3K4me1, H3K4me2, H3K4me3, and H3 antibodies, with additional loading marks (mk). *n* = 10 per genotype. **B** Verification of reduced H3K4me2 using immunohistochemistry in the CA1 (*p < 0.05, *t*_(12)_ = 2.37, 2-tailed), CA3 (*p < 0.05, *t*_(12)_ = 2.81, 2-tailed) and DG (*p < 0.05, *t*_(12)_ = 2.82, 2-tailed) region of the hippocam*p*us of TG relative to WT mice. The photomicrographs show representative immunofluorescence H3K4me2 staining in the CA1, CA3 and DG regions. *n* = 7 per genotype. **C** Western blot analysis of LSD1, normalized to H3 housekeeping control. The photograph shows a representative Western blot using LSD1 and H3 antibodies, with additional loading marks (mk). *n* = 10 per genotype. **D** Enzymatic activity of LSD1, as measured with a fluorometric quantification assay; *p < 0.05 (*t*_(18)_ = 2.16, 2-tailed); *n* = 10 per genotype. All scatter plots show individual mice with overlaid group means ± s.e.m. **E** Simplified schematic illustration of the proposed mechanism underlying altered H3K4 methylation dynamics in mice expressing HERV-W ENV relative to controls. Concurrent to the downregulation of H3K4 methyltransferases (H3K4 MTs; also see Fig. 3), increased enzymatic activity of LSD1 in HERV-W ENV mice may shift the methylation dynamics towards increased and decreased H3K4me1 and H3K4me2/me3, respectively.

The RNAseq analyses demonstrated that transgenic expression of HERV-W ENV induces transcriptional alterations in molecular systems that regulate histone methylation. Specifically, *Kmt2a*, *Kmt2b, Kmt2c*, *Kmt2d*, and *Setd1a*, which were found to be downregulated in the hippocampus of TG mice relative to WT controls (Fig. 2; Supplementary Table 5), are genes that encode histone methyltransferases catalyzing the methylation of H3K4 to H3K4me1, H3K4me2, and/or H3K4me3 [71–73]. In view of the transcriptional downregulation of these methyltransferases in the hippocampus of TG mice (Fig. 2; Supplementary Table 5), we hypothesized that transgenic expression of HERV-W ENV alters hippocampal H3K4 methylation. In support of this hypothesis, Western blot analyses revealed reduced levels of H3K4me2 and H3K4me3 in the hippocampus of TG mice relative to WT controls, whereas H3K4me1 levels were increased in the former compared to the latter group (Fig. 4A). We further verified alterations in H3K4 methylation states using immunohistochemistry, confirming reduced H3K4me2 immunoreactivity in the hippocampus of TG mice relative to WT controls (Fig. 4B).

To examine whether these H3K4 methylation changes are associated with altered demethylase activity, we measured the protein levels and enzymatic activity of LSD1 in the hippocampus of TG and WT mice. We focused on LSD1, a flavin-dependent monoamine oxidase primarily removing methyl groups from H3K4me2 to H3K4me1 [74, 75]. As shown in Fig. 4C, hippocampal protein levels of LSD1 were not different between TG and WT mice. There was, however, a significant increase in the enzymatic activity of LSD1 in TG mice relative to WT controls (Fig. 4D). Taken together, these findings demonstrate that transgenic expression of HERV-W ENV leads to a concomitant downregulation of H3K4 methyltransferase genes (Fig. 2; Supplementary Table 5), alterations in H3K4 methylation (Fig. 4A), and increased LSD1 activity. The latter potentially contributes to the shift in the methylation dynamics towards increased and decreased H3K4me1 and H3K4me2, respectively (Fig. 4E).

### Restoration of altered H3K4 methylation and neuropsychiatric phenotypes through pharmacological inhibition of LSD1

Reduced H3K4me2 methylation has been causally linked to the emergence of behavioral and cognitive deficits in other preclinical mouse models relevant to psychiatric and neurodevelopmental disorders, including models that are based on genetic deficiency of *Shank3* [41], *Setd1a* [42], and *Kmt2c* [76]. Importantly, restoring deficient H3K4me2 through pharmacological inhibition of histone demethylases, such as LSD1, was found to be efficient in normalizing schizophrenia- and ASD-related phenotypes in these models [41, 42, 76]. Because we identified a concomitant reduction in mRNA expression of histone methyltransferases (*Kmt2a*, *Kmt2b*, *Kmt2d*, and *Setd1a*) and H3K4me2 protein, we investigated whether pharmacological inhibition of LSD1 normalizes the epigenetic, behavioral, and cognitive deficits in mice expressing HERV-W ENV. To this end, we treated WT and TG mice chronically with ORY-1001, which is a brain-penetrable and selective inhibitor of LSD1 that increases H3K4me2 [39, 41, 42]. Chronic vehicle (VEH) administration served as negative control treatment.

**Fig. 5:**
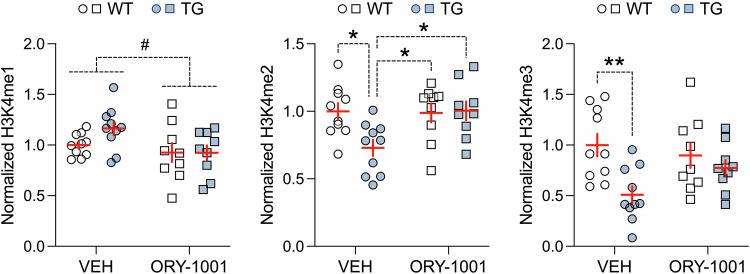
Normalization of abnormal H3K4 methylation in the adult hippocampus through pharmacological inhibition of LSD1. Male transgenic CAG^HERV-Wenv^ mice (TG) and wild-type (WT) littermates were treated with the LSD1 inhibitor, ORY-1001, or corresponding vehicle (VEH). The scatter plots (with overlaid means ± s.e.m.) depict the Western blot analysis of H3K4 mono-methylation (H3K4me1), di-methylation (H3K4me2) and tri-methylation (H3K4me3), normalized to H3 housekeeping control, in the hippocampus of WT and TG mice. *p < 0.05 and **p < 0.01, based on Tukey’s post-hoc test following a significant 2-way interaction between genotype and treatment in ANOVA of H3K4me2 (*F*_(1,34)_ = 4.89, *p* < 0.05) and H3K4me3 (*F*_(1,34)_ = 4.18, *p* < 0.05). ^#^*p* = 0.064, reflecting the main effect of treatment at statistical trend level (*F*_(1,34)_ = 3.49). *n*(WT/VEH) = 10 mice, *n*(TG/VEH) = 10 mice, *n*(WT/ORY-1001) = 9 mice, and *n*(TG/ORY-1001) = 9 mice.

We found that ORY-1001 treatment in mice expressing HERV-W ENV fully restored the decrease in hippocampal H3K4me2, such that TG mice receiving ORY-1001 displayed H3K4me2 levels that were equivalent to those measured in VEH-treated WT mice (Fig. 5). In addition, ORY-1001 treatment led to a partial normalization of reduced H3K4me3 levels in mice expressing HERV-W ENV (Fig. 5). On the other hand, H3K4me1 levels were largely spared by the pharmacological intervention, albeit ORY-1001 led to a trend towards reducing H3K4me1 levels in TG mice (Fig. 5). These data suggest that pharmacological inhibition of LSD1 with its specific inhibitor, ORY-1001, is particularly effective in restoring abnormal H3K4me2 marks.

**Fig. 6:**
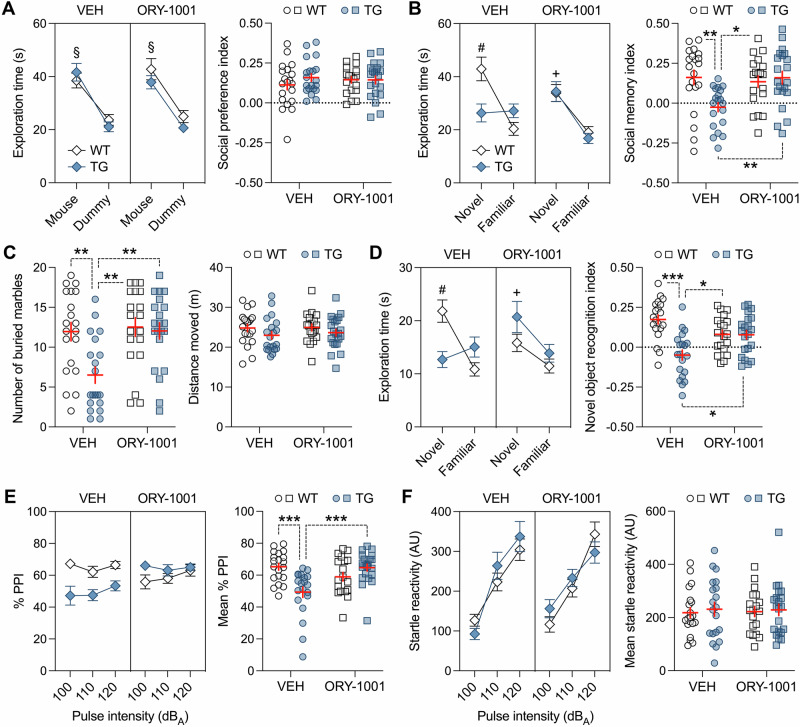
Normalization of adult behavioral and cognitive deficits in HERV-W ENV mice through pharmacological inhibition of LSD1. Male transgenic CAG^HERV-Wenv^ mice (TG) and wild-type (WT) littermates were treated with the LSD1 inhibitor, ORY-1001, or corresponding vehicle (VEH). **A** The line plot depicts the means ± s.e.m. of time spent with the unfamiliar mouse or dummy object, whereas the scatter plot (with overlaid means ± s.e.m.) shows the social preference index (values > 0 represent a preference toward the unfamiliar mouse) in phase 1 of the social interaction test. ^§^*p* < 0.001, reflecting the significant main effect of object (*F*_(1,76)_ = 87.53) in repeated-measures ANOVA. **B** The line plot depicts the means ± s.e.m. of time spent with the novel or familiar mouse, whereas the scatter plot (with overlaid means ± s.e.m.) depicts the social memory index (values > 0 represent a preference toward the novel mouse) in phase 2 of the social interaction test. ^#^*p* < 0.001 and ^+^*p* < 0.001, reflecting the significant difference between novel and familiar mouse exploration in VEH-treated WT mice and in ORY-1001-treated WT or TG mice, respectively, based on Šidák post-hoc test after the presence of a significant 3-way interaction between genotype, object and treatment (*F*_(1,76)_ = 23.68, *p* < 0.001) in repeated-measures ANOVA. *p < 0.05 and **p < 0.01, based on Tukey’s post-hoc test following a significant 2-way interaction between genotype and treatment (*F*_(1,76)_ = 7.61, *p* < 0.01) in ANOVA. **C** Number of buried marbles and distance moved during the marble burying test. **p < 0.01, based on Tukey’s post-hoc test following a significant 2-way interaction between genotype and treatment (*F*_(1,76)_ = 5.21, *p* < 0.05) in ANOVA. **D** The line plot depicts the means ± s.e.m. of time spent with the novel or familiar object, whereas the scatter plot (with overlaid means ± s.e.m.) depicts the novel object recognition memory index (values > 0 represent a preference toward the novel object) in the novel object recognition test. ^#^*p* < 0.001 and ^+^*p* < 0.01, reflecting the significant difference between novel and familiar object exploration in VEH-treated WT mice and in ORY-treated WT or TG mice, respectively, based on Šidák multiple comparison post-hoc test after the presence of a significant 3-way interaction between genotype, object and treatment (*F*_(1,76)_ = 19.24, *p* < 0.001) in repeated-measures ANOVA; *p < 0.05 and ***p < 0.001, based on Tukey’s post-hoc test following a significant 2-way interaction between genotype and treatment (*F*_(1,76)_ = 4.32, *p* < 0.05) in ANOVA. **E** The line plots depict the means ± s.e.m. of % PPI averaged across different pulse intensities (100, 110 and 120 dB_A_); the scatter plot (with overlaid means ± s.e.m.) depicts the mean % PPI across all prepulse and pulse intensities. ***p < 0.001, based on Tukey’s post-hoc test following a significant 2-way interaction between genotype and treatment (*F*_(1,76)_ = 17.05, *p* < 0.001) in repeated-measures ANOVA. **F** The line plots depict the means ± s.e.m. of startle reactivity (in arbitrary units, AU) to 100-, 110- and 120-dB_A_ pulse stimuli, whereas the scatter plot (with overlaid means ± s.e.m.) shows the mean startle reactivity (in AU) across all pulse intensities. *n*(WT/VEH) = 20 mice, *n*(TG/VEH) = 20 mice, *n*(WT/ORY-1001) = 19 mice, and *n*(TG/ORY-1001) = 21 mice.

Importantly, ORY-1001 treatment was also highly effective in normalizing behavioral and cognitive deficits in mice with transgenic expression of HERV-W ENV (Fig. 6). Specifically, we found that the pharmacological intervention restored the deficits in social memory in the social interaction test (Fig. 6B), repetitive behavior in the marble burying test (Fig. 6C), object memory in the novel object recognition test (Fig. 6D), and sensorimotor gating in the PPI test (Fig. 6E). These therapeutic effects emerged selectively in TG mice and were not accompanied by drug-induced alterations in WT mice (Fig. 6). Social approach behavior in the social interaction test (Fig. 6A), locomotor activity in the marble burying test (Fig. 6C), and acoustic startle reactivity (Fig. 6F) were not altered by ORY-1001 treatment. Taken together, these data demonstrate that pharmacological inhibition of LSD1 had selective therapeutic effects on behavioral and cognitive impairments without influencing functions that were unaffected by transgenic expression of HERV-W ENV under basal conditions.

## Discussion

Using a mouse model of human endogenous retroviral expression, our data show that constitutive expression of HERV-W ENV induces behavioral and cognitive deficits pertaining to psychiatric and neurodevelopmental disorders. These deficits include impairments in social memory, object recognition memory, and sensorimotor gating, which are present especially in psychosis-related disorders and ASD [50–56]. Interestingly, we also revealed lower amounts of repetitive behavior in the marble burying test, which is consistent with previous findings in the *Shank1* and *Shank3B* mutant mouse models of ASD [48, 49]. Further supporting the translational relevance of our mouse model to psychiatric and neurodevelopmental disorders, our study identified a transcriptional downregulation of several genes implicated in schizophrenia and ASD, including *Setd1a* [62–64], *Cacna1g* [64, 67], *Ank3* [68], *Shank1* [69, 70], and *Shank3* [69, 70], in mice expressing HERV-W ENV. These transcriptional changes emerged specifically in the hippocampus, but not in the PFC, suggesting that hippocampal regions may be more susceptible to HERV-W ENV expression than prefrontal regions, at least with regards to transcriptomic alterations in adulthood. Unlike previous in vitro studies, which found that acute exposure to recombinant HERV-W ENV protein induced glial activation in rat glial or mixed cell cultures[14, 24, 77], our RNA-seq analyses did not reveal transcriptomic alterations indicative of glial activation or neuroinflammation. Interestingly, however, we previously observed activation of astrocytes and microglia in HERV-W ENV-expressing mice only under additional environmental exposures, such as demyelinating cuprizone treatment, but not under baseline conditions [14]. Thus, ubiquitous HERV-W ENV expression may prime or polarize glial cells toward heightened and potentially neurotoxic responses, which may only become evident with additional environmental triggers [6, 14].

Our study further suggests that the pathological effects of HERV-W ENV expression are amenable to therapeutic interventions. In support of this hypothesis, we found that pharmacological inhibition of the histone demethylase, LSD1, normalizes the behavioral and cognitive deficits in adult mice expressing HERV-W ENV. Based on previous preclinical studies in mice [41, 42], we used ORY-1001 as pharmacological inhibitior of LSD1 in our model. ORY-1001 was originally developed for its potential to mitigate altered histone methylation associated with acute leukemia [39]. However, beyond leukemia, ORY-1001 and other LSD1 inhibitors are increasingly tested in preclinical models relevant to neurodevelopmental and psychiatric disorders, including schizophrenia and ASD. For instance, ORY-1001 has shown efficacy in normalizing ASD-related [41] and schizophrenia-related [42] symptoms in *Shank3*-deficient and *Setd1a*-deficient mice, respectively. Additionally, other LSD1 inhibitors, such as DDP-38003 and TAK-418, have been reported to ameliorate ASD-like behaviors in mouse models of maternal immune activation and 7q11.23 deletion [78, 79]. In these studies, LSD1 inhibitors like ORY-1001 effectively rescued various ASD- and/or schizophrenia-related behavioral and cognitive deficits without producing detectable side effects in control animals. Our findings align with these previous observations, further supporting the potential of LSD1 inhibitors to ameliorate a range of behavioral and cognitive dysfunctions associated with neurodevelopmental and psychiatric disorders.

As recently reviewed [80], a primary rationale for testing LSD1 inhibitors, such as ORY-1001, in preclinical models of neurodevelopmental and psychiatric disorders stems from observations of reduced H3K4me2 and/or H3K4me3 methylation in the brains of individuals with schizophrenia and ASD [41, 81, 82]. Here, we report that adult mice with HERV-W ENV expression exhibit a similar reduction in H3K4me2 and H3K4me3 methylation. At the same time, however, TG animals showed elevated levels of H3K4me1 in the hippocampus. The diametrically opposite changes in H3K4me1 *versus* H3K4me2 and H3Kme3 are likely to be explained by the dynamics of histone methylation and demethylation processes [83–86]. In support of this hypothesis, we found increased enzymatic activity of LSD1, which primarily removes histone H3K4me2 to H3K4me1 and H3Kme0 [74, 75]. Thus, increased LSD1 activity in mice expressing HERV-W ENV may readily shift the methylation dynamics towards increased and decreased H3K4me1 and H3K4me2, respectively. Consequently, inhibiting LSD1 enzymatic activity with ORY-1001 was expected to shift the methylation balance towards normalized H3K4me2 levels. Our findings supported this hypothesis, showing that ORY-1001 treatment in HERV-W ENV mice fully restored H3K4me2 levels to those observed in WT control mice. Consistent with our findings, ORY-1001 has also been shown to restore H3K4me2 levels in the brains of *Shank3*-deficient and *Setd1a*-deficient mice [41, 42], suggesting that normalization of reduced H3K4me2 levels may be a key mechanism by which ORY-1001 alleviates ASD- and schizophrenia-related dysfunctions.

In a broader context, our findings have relevance for advancing our understanding of the association between abnormal expression of HERVs and psychiatric and neurodevelopmental disorders. Even though increased HERV-W expression has long been implicated in mental illnesses, including schizophrenia, bipolar disorder and ASD [25–34], this association has thus far been supported mostly by correlative evidence. Our findings provide preclinical evidence for a causal relationship between HERV-W ENV expression and pathological changes in specific behavioral, cognitive and molecular domains with translational relevance to psychiatric and neurodevelopmental disorders. Notably, our findings appear particularly relevant for a subgroup of patients who display overt elevations in HERV-W expression. For example, it is estimated that up to ~40% and ~30% of patients with schizophrenia and bipolar disorder, respectively, show elevated levels of HERV-W ENV [30]. This proportion is likely to be higher during post-acute phases of certain infections, such as SARS-CoV2 [31, 87], *Toxoplasma gondii* [29] and EBV [88, 89], which are known to re-activate HERV-W from a dormant, non-activated to activated state [5, 6, 12]. Interestingly, infections with these pathogens have been widely implicated in the etiology and pathophysiology of psychiatric and neurodevelopmental disorders [90–97], even without the explicit consideration of a possible link to HERV-W re-activation. In this regard, our findings may encourage further investigations in the possible associations between infections, HERV-W re-activation and psychiatric and neurodevelopmental disorders.

We acknowledge several limitations in our study. First, all findings described herein are based on male mice only. The reason for selecting the male sex was that the MSRV-pV14-env transgene was inserted into the X-chromosome [14, 37]. As a result of the X-chromosome insertion, we paired WT males with heterozygous TG females to obtain male hemizygous TG mice and WT littermates for inclusion in our study. Because sex differences are highly relevant for neurodevelopmental and psychiatric disorders [98, 99], future studies involving different breeding schemes will be needed to assess the functional impact of HERV-W ENV expression in female mice. Second, our data are based on constitutive expression of HERV-W ENV, and therefore, our study was not designed to examine possible effects of its expression during specific developmental windows. While we deem our findings an important first step to causally link HERV-W ENV expression to the disruption of brain functions with relevance to neurodevelopmental and psychiatric disorders, the future establishment of conditional transgenic mouse models with time-restricted and region-specific expression of human-specific endogenous retroviruses will be required to identify sensitive windows and vulnerable brain areas that are particularly affected by HERV-W ENV expression. Third, our ORY-1001 administration regimen was adapted from a previous study, which showed that this LSD1 inhibitor rescued working memory deficits in *Setd1a*-deficient mice [42]. Similar to our study, ORY-1001 was administered daily to *Setd1a*-deficient and WT mice at a dose of 0.01 mg/kg over a two-week period prior to a non-match-to-sample T-maze working memory task, with treatment continuing throughout the T-maze testing phase [42]. Notably, this administration protocol did not allow us to determine whether the therapeutic effects of ORY-1001 were attributable to the cumulative impact of repeated drug dosing or to its acute effects during testing. This issue should be further examined in future studies. Additionally, given the adult emergence of behavioral and cognitive deficits in our model of HERV-W ENV expression, it would be valuable to investigate whether early interventions that are based on adolescent treatment with LSD1 inhibitors can effectively prevent the subsequent emergence of adult deficits. Finally, additional molecular analyses will be needed to establish how the identified epigenetic changes in histone methylation influence the transcriptomic landscape in response to HERV-W ENV expression. Our findings, along with those from prior studies [41, 42, 76], suggest that the normalization of reduced H3K4me2 levels is a feasible mechanism through which ORY-1001 alleviates ASD- and schizophrenia-related dysfunctions. While this mechanism does not offer comprehensive insights into the precise biological processes influenced by ORY-1001, it does open avenues for future exploration of the specific neurobiological pathways modulated by HERV-W ENV expression and its normalization via LSD1 inhibitors.

Despite these limitations, we conclude that the expression of HERV-W ENV has the capacity to disrupt various behavioral and cognitive functions and to alter the brain transcriptome in a manner that is highly relevant to neurodevelopmental and psychiatric disorders. Moreover, our study identified epigenetic pathways that may offer avenues for pharmacological interventions against behavioral and cognitive deficits induced by increased HERW-W expression. In this regard, it should be noted that LSD1 inhibitors are currently under investigation in preclinical studies [78, 100] and clinical trials (EudraCT No.: 2021-000350-26) for the treatment of psychosis-related disorders and ASD. The experimental model system described here offers unique opportunities to further investigate the therapeutic benefits of LSD1 inhibitors for these disorders in the context of HERW-W expression and beyond.

## Supplementary information

Supplemental information file

## Data Availability

All data needed to evaluate the conclusions in the paper are present in the paper and the Supplementary Information. The RNA-seq data discussed in this publication have been deposited and are accessible at Zenodo through accession number 10.5281/zenodo.14184511 (https://zenodo.org/records/14184511).
