# Supplementary material for: Recapitulation and reversal of neuropsychiatric phenotypes in a mouse model of human endogenous retrovirus type W expression
Source: Mol Psychiatry. 2025 Mar 18;30(7):3325–37. doi: 10.1038/s41380-025-02955-9 (PMC12185329; doi:10.1038/s41380-025-02955-9)
Supplement: Supplementary file 1 — Supplemental information file [file 41380_2025_2955_MOESM1_ESM.pdf]

SUPPLEMENTARY INFORMATION

Recapitulation and reversal of neuropsychiatric phenotypes in a mouse model of human endogenous retrovirus type W expression

Felisa Herrero[1], Celine Heeb[1], Michelle Meier[1], Han-Yu Lin[1], Flavia S. Mueller[1], Sina M. Schalbetter[1], Joel Gruchot[2], Ulrike Weber-Stadlbauer[1], Tina Notter[3,4], Hervé Perron[5,6], Patrick Küry[2,7], Urs Meyer[1,4]

[1]Institute of Veterinary Pharmacology and Toxicology, University of Zurich, Zurich, Switzerland.
[2]Department of Neurology, Medical Faculty, Heinrich-Heine-University Düsseldorf, Düsseldorf, Germany.
[3]Institute of Pharmacology and Toxicology, University of Zurich, Zurich, Switzerland.
[4]Neuroscience Center Zurich, University and ETH Zurich, Zurich, Switzerland.
[5]GeNeuro, 18, chemin des Aulx, 1228 Plan-les-Ouates, Geneva, Switzerland.
[6]Université de Lyon-UCBL, Lyon, France.
[7]Department of Neurology, Inselspital, University Hospital and University of Bern, Bern, Switzerland.

Correspondence:
Dr. Urs Meyer (PhD)
Institute of Veterinary Pharmacology and Toxicology, University of Zurich
Winterthurerstrasse 260, CH-8057, Zurich, Switzerland.
E-mail: urs.meyer@vetpharm.uzh.ch
Tel.: +41 44 635 88 44

SUPPLEMENTARY METHODS

Animals

Transgenic C57BL6/J;129P2/Ola-Hprt mice (referred to as $CAG^{HERV\text{-}Wenv}$ TG mice) and wild-type (WT) littermates were used throughout the studies, as described in the main text. Male hemizygous TG mice and WT littermates were generated by breeding WT males with heterozygous TG females. All animals were weaned on postnatal day (PND) 21, at which point ear biopsies for genotyping were taken. In short, biopsy tissue was lysed with 75µl lysis buffer (25mM NaOH and 0.2mM EDTA (5ml of 5M NaOH, 2ml of 01M EDTA, ddH_2O to 1L, pH12)) at 96°C for 20min, after which 75µl of neutralising buffer (40mM Tris-HCl (6.3g Tris-HCL, ddH_2O to 1L, pH 5)) was added. DNA concentration was measured utilizing a Nanodrop (DeNovix DS-11+ spectrophotometer, Labgene Scientific SA, Switzerland) and diluted to a concentration of 4ng/µl. For the detection of *Hprt* WT and HERV-W ENV TG alleles, the following primer pairs were used: WT_forward: 5′-TGTCCTTAGAAAACACATATCCAGGGTTTAGG-3'; WT_reverse: 5′-CTGGCTTAAAGACAACATCTGGGAGAAAAA-3;' TG_forward: 5′-TTTACTCCTCTTTGG ACCCT-3'; TG_reverse: 5′-ATCTGGGGTTCCATTTGAAG-3'. Glyceraldehyde 3-phosphate dehydrogenase (GAPDH) was used as housekeeping gene, with the following primer pairs: GAPDH_forward: 5'-AACGACCCCTTCATTGAC-3' and GAPDH_reverse: 5'- CTCCACGACAT ACTCAGCAC-3'. The real-time PCR reaction included 20 ng DNA, 0.3 µl of forward and reverse primers, and 5 µl of SsoAdvanced™ Universal SYBR® Green Supermix (Biorad, Hercules, California, USA). Thermal cycling was initiated at 95°C for 30 s, followed by 40 PCR cycles (10s at 95°C; 20 s at 60°C) and a melt-curve analysis (65-95°C, 0.5°C increments at 5 sec/step). The genotype of the animals was determined by the presence or absence of target gene expression.

All animals were kept in a temperature- and humidity-controlled (21±1°C, 55±5%), specific pathogen free (SPF) holding facility under a reversed light-dark cycle (lights off: 09.00-21.00) and were maintained under *ad libitum* standard rodent chow and water. The animals were group-

housed (3-5 animals per cage) in individually ventilated cages (IVCs). **Supplementary Table 1** provided an overview of the animal cohorts used in this study.

All procedures described in the present study had been previously approved by the Cantonal Veterinarian's Office of Zurich, Switzerland, and by the review board of the state government Landesamt für Natur, Umwelt und Verbraucherschutz Nordrhein-Westfalen, North-Rhine Westphalia, Germany. All efforts were made to minimize the number of animals used and their suffering.

Cohort	Sample size	Experimental series
1a	WT = 27<br>TG = 27	Behavioral and cognitive testing of adult animals (1. open field test; 2. light-dark box test; 3. marble burying test; 4. social interaction test; 5. novel object recognition test; 6. PPI test)
1b	WT = 15<br>TG = 17	Behavioral and cognitive testing of adolescent animals (1. social interaction test; 2. novel object recognition test; 3. PPI test)
2a	WT = 3<br>TG = 3	Next-generation RNA sequencing of bulk prefrontal cortex and hippocampus
2b	WT = 11<br>TG = 11	Gene expression analysis of selected candiate genes in bulk hippocampal tissue using quantitative real-time PCR
2c	WT = 6<br>TG = 6	Gene expression analysis of selected candiate genes in dorsal and ventral hippocampal tissue using quantitative real-time PCR
3a	WT = 10<br>TG = 10	Western blot analyses of H3, H3K4me1, H3K4me2, H3K4me3, and LSD1 in bulk hippocampal tissue
3b	WT = 7<br>TG = 7	Immunohistochemical analyses of H3K4me2, VGLUT1/PSD-95, and VGAT/Gephyrin in CA1, CA3 and DG subregions of the hippocampus
3c	WT = 10<br>TG = 10	Measurement of enzymatic activity of LSD1 in bulk hippocampal tissue
4	WT (VEH) = 10<br>TG (VEH) = 10<br>WT (ORY-1001) = 9<br>TG (ORY-1001) = 9	Western blot analyses of H3, H3K4me1, H3K4me2, H3K4me3, in bulk hippocampal tissue after chronic treatment with vehicle (VEH) or ORY-1001
5	WT (VEH) = 20<br>TG (VEH) = 20<br>WT (ORY-1001) = 19<br>TG (ORY-1001) = 21	Behavioral and cognitive testing (1. open field test; 2. marble burying test; 3. social interaction test; 4. novel object recognition test; 5. PPI test) after chronic treatment with vehicle (VEH) or ORY-1001

Supplementary Table 1. Summary of the animal cohorts used in this study. Male transgenic $CAG^{HERV\text{-}Wenv}$ mice (TG) and wild-type (WT) littermates were used in all investigations. The table specifies the sample size of each cohort and their allocation to the experimental series of interest.

Open field test

A standard open field test was used to asses basal locomotor activity and innate anxiety-like behavior in a novel environment.[1] The test was conducted in 4 identical square arenas (40 × 40 cm) surrounded by walls (35 cm high). The apparatus was made of white Plexiglas and was located in a testing room under dim lighting (28 lx in the center zone, 20 lx in the corner of the apparatus). A digital camera was mounted directly above the four arenas. Images were captured at a rate of 5 Hz and transmitted to a PC running the Ethovision (Noldus Information Technology, The Netherlands) tracking system. The animals were gently placed in the center of the arena and allowed to freely explore for the duration of 10 min. For the purpose of data collection, the arena was conceptually partitioned into two areas: a center zone (measuring 15 ×15 cm^2) in the middle of the area, and a peripheral zone occupying the remaining area. The dependent measures were total distance moved (m) in the entire arena, number of center zone visits, and time (s) spent in the center zone.

Light-dark box test

A light–dark box test was used as a second test to measure innate anxiety-like behavior.[1] The apparatus consisted of four identical multi-conditioning boxes (Multi Conditioning System, TSE Systems, Bad Homburg, Germany), each containing a dark (1 lx) and a bright (100 lx) compartment. The two compartments were separated from each other by a dark plexiglass wall with an integrated, electrically controlled door. To start a trial, each mouse was placed in the dark compartment. After 5 s the door automatically opened, allowing access to both the dark and bright compartment for 10 min. The measurements collected from this test included the total distance moved (m), distance moved in the light compartment (m), and time (s) spent in the light compartment.

Marble burying test

To assess repetitive, compulsive-like behaviors, we used the marble burying test,[2,3] which is commonly used in preclinical research of ASD and other neurodevelopmental disorders.[4,5] The

test was performed as described by Vuillermot et al.[6] Clean Plexiglas cages (27 × 16.5 × 12.5 cm) were filled with a 2-cm layer of chipped cedar wood bedding. The animals were first habituated to this cage for 10 min and then were returned to the home cage. Twenty colored glass marbles (15 mm diameter) were then gently laid on top of the bedding, equidistant from each other in a 4 × 5 arrangement. Animals were placed back into the testing cage, and the number of marbles buried (criteria for a buried marble was >75% marble covered by bedding material) in 10 min recorded. In addition, the total distance moved (m) in the test apparatus was measured using a digital camera that was mounted directly above the apparatus, which captured images at a rate of 5 Hz and transmitted to a PC running the Ethovision (Noldus Information Technology, The Netherlands) tracking system.

Social interaction test

Sociability and social recognition memory were measured using a social interaction test consisting of two phases, as described recently.[7] The apparatus was made of a modified Y-maze consisting of an opaque acrylic glass, which contained three identical arms (50 cm [length] by 9 cm [width]) surrounded by 10-cm-high Plexiglas walls. The three arms radiated from a central triangle (8 cm on each side) and were spaced 120° from each other. Two of the three arms contained rectangular wire grid cages (13 cm [length] by 8 cm [width] by 10 cm [height]), with metal bars horizontally and vertically spaced 9 mm apart. The third arm did not contain a metal wire cage and served as the start zone (see below). The apparatus was located in an experimental testing room under dim diffused lighting (~20 lx as measured in the individual arms).

Phase 1: This phase served as a test for sociability. During this phase, one metal wire cage contained an unfamiliar WT mouse ("live mouse"), whereas the other wire cage contained an inanimate object ("dummy object"). The latter was a black scrunchie made of velvet material. The allocation of the live mouse and "dummy" to the two wire cages was counterbalanced across experimental groups. To begin a trial, the test animal was introduced at the end of the start arm and was allowed to freely explore all three arms for 5 min. Behavioral observations were made

by an experimenter who was blinded to the experimental conditions in the form of numerical codes. Social interaction was defined as a nose contact within a 2-cm interaction zone. For each test animal, a social preference index was calculated by the following formula: [time spent with the mouse/(time spent with the inanimate dummy object + time spent with the mouse)] − 0.5. The social preference index was used to compare the relative exploration time between the unfamiliar mouse and the inanimate dummy object, with values > 0 signifying a preference toward the unfamiliar mouse. In addition, the absolute times spent with the unfamiliar mouse and the inanimate dummy object were analyzed. On completion of phase 1, the animal was removed and kept in a holding cage for 5 min until the start of the next phase.

Phase 2: This phase served as a test for social recognition memory. During this phase, another unfamiliar WT mouse, which was referred to as the “novel mouse,” replaced the inanimate dummy object. The other cage contained the “familiar mouse” previously used in phase 1 (see above) of the test. The allocation of the novel mouse and familiar mouse to the two wire cages was counterbalanced across experimental groups. To start phase 2, the test animal was introduced at the end of the start arm and was allowed to freely explore all three arms for 5 min. Behavioral observations for social interaction were scored as described before. For each test animal, a social memory index was calculated by the following formula: [time spent with the novel mouse/(time spent with the novel mouse + time spent with the familiar mouse)] − 0.5. The social memory index was used to compare the relative exploration time between the familiar and novel mice, with values > 0 signifying a preference toward the novel mouse. In addition, the absolute times spent with the familiar and novel mice were analyzed.

Novel object recognition test

The novel object recognition test was used to assess short-term declarative memory,[8,9] which is disrupted in numerous psychiatric disoders.[10] The test apparatus consisted of an open field as described above, with minor modifications (see below). The test procedure consisted of three consecutive phases, which were each separated 60 min apart.

Habituation phase: To habituate the animals to the test apparatus, the animals were gently placed in the center of the open-field apparatus and allowed to freely explore the arena for 10 min. They were then removed from the apparatus and kept in a holding room for 60 min before the start of the sample phase.

Sample phase: For this phase, a first pair of objects (aluminium hairspray can, glass bottle filled with sand, or a LEGO® Duplo brick pile) were placed in the open field arena in opposing corners approximately 5 cm from the walls. The animals were placed into the center of the arena and were allowed to freely explore the objects for 10 min. They were then removed from the apparatus again and kept in a holding room for another 60 min before the start of the test phase.

Test phase: For this phase, a novel object, different from either of the two initial objects (familiar object), replaced one of the objects from the sample phase. Animals were placed into the center of the arena again and allowed to freely explore the objects for 10 min. For each animal, a novel object recognition index was calculated by the following formula: [time spent with novel object/(time spent with novel object + time spent with familiar object)] − 0.5. The novel object recognition index was used to compare the animals' capacity to discriminate the novel versus familiar object, with values > 0 representing a preference toward the novel object. In addition, the absolute times spent with the familiar and novel objects were analyzed.

Prepulse inhibition (PPI) of the acoustic startle reflex

Sensorimotor gating was assessed using the paradigm of prepulse inhibition (PPI) of the acoustic startle reflex. PPI of the acoustic startle reflex refers to the reduction in startle reaction in response to a startle-eliciting pulse stimulus when it is shortly preceded by a weak prepulse stimulus.[10,11] This form of sensorimotor gating is known to be impaired in schizophrenia and other psychiatric disorders.[10,11]

The apparatus consisted of four startle chambers for mice (San Diego Instruments, San Diego, CA, USA) and has been fully described elsewhere.[12,13] In the demonstration of PPI, the animals were presented with a series of discrete trials comprising a mixture of four trial types. These included pulse-alone trials, prepulse-plus-pulse trials, prepulse-alone trials, and no-

stimulus trials in which no discrete stimulus other than the constant background noise was presented. The pulse and prepulse stimuli used were in the form of a sudden elevation in broadband white noise level (sustained for 40 and 20 ms, respectively) from the background (65 dB_A), with a rise time of 0.2–1.0 ms. In all trials, three different intensities of pulse (100, 110, and 120 dB_A) and three intensities of prepulse (71, 77, and 83 dB_A, which corresponded to +6, +12, and +18 dB_A above background, respectively) were used. The stimulus-onset asynchrony of the prepulse and pulse stimuli on all prepulse-plus-pulse trials was 100 ms (onset-to-onset).

The protocol used for the PPI test was extensively validated before.[12,14] A session began with the animals being placed into the Plexiglas enclosure. They were acclimatized to the apparatus for 2 min before the first trial began. The first 6 trials consisted of 6 startle-alone trials; such trials served to habituate and stabilize the animals' startle response and were not included in the analysis. Subsequently, the animals were presented with 10 blocks of discrete test trials. Each block consisted of the following: three pulse-alone trials (100, 110, or 120 dB_A), 3 prepulse-alone trials (+6, +12, or +18 dB_A above background), 9 possible combinations of prepulse-plus-pulse trials (3 levels of pulse × 3 levels of prepulse), and one no stimulus trial. The 16 discrete trials within each block were presented in a pseudorandom order, with a variable interval of 15 s on average (ranging from 10 to 20 s). For each of the 3 pulse intensities (100, 110, or 120 dB_A), PPI was indexed by percent inhibition of the startle response obtained in the pulse-alone trials by the following expression: 100% × (1 − [mean reactivity on prepulse-plus-pulse trials/mean reactivity on pulse-alone trials]), for each animal, and at each of the three possible prepulse intensities (+6, +12, or +18 dB_A above background). In addition to PPI, reactivity to pulse-alone trials was analyzed to assess possible effects on acoustic startle reactivity *per se*.

ORY-1001 treatment

ORY-1001 (Cayman chemicals, Michigan, USA, product number: 19136) or vehicle (VEH) was administered *per os* using the micropipette-guided drug administration (MDA) method, a stress-free alternative to oral gavage for chronic *per os* treatments in mice.[15,16] The MDA method is based on the presentation of a palatable solution consisting of sweetened condensed milk mixed

with water, which motivates the animals to consume vehicle and/or drug solutions voluntarily. Because of its palatable nature, mice quickly (typically less than 3 days) learn to freely drink the sweetened condensed milk solution from conventional micropipettes in the presence of the experimenter.[15,16] Thus, the MDA technique allows administration of substances without the need for a full restraint or invasive manipulations, thereby minimizing the stressful impact on the experimental animals. At the same time, it allows the experimenter to control for the dosing and timing of the administered substance.[15,16]

The basic principles of the MDA technique have been described elsewhere.[15,16] Ory-1001 vials were kept at -20°C until use, according to manufacture indications, and used to prepare fresh drug solutions every second day. The drug was diluted to a final concentration of 0.01mg/kg in a 2ml/kg administration volume of 4:10 sweetened condensed milk (Migros Kondensmilch; Migros) mixed with water, as described in Scarborough et al.[15] When the animals reached 10 weeks of age, WT and TG groups were randomly assigned to receive either VEH or ORY treatment. ORY-1001 or VEH was given daily for two weeks prior to the commencement of behavioral and cognitive testing, after which it was maintained throughout the entire testing period. During the behavioral testing phase, the drug and the corresponding vehicle were always given after testing, that is, at the end of the reversed light-dark cycle between 19:00 and 21:00.

Next-generation RNA sequencing and gene network analysis

We performed next-generation RNA sequencing of prefrontal and hippocampal samples from adult (12-weeks old) WT and TG mice. Following decapitation of the animals and subsequent brain extraction, samples of the prefrontal cortex (PFC; containing anterior cingulate, prelimbic and infralimbic subregions) and whole hippocampi were isolated and stored at −80°C until further use, as described before.[17]

Total RNA was extracted from prefrontal and hippocampal samples using the SPLIT RNA extraction kit (Lexogen, Austria) following the manufacturer's recommendations and was sent to the Functional Genomics Center in Zurich (FGCZ) for quality control and RNA sequencing. The quality of the isolated RNA was determined with a Fragment Analyzer (Agilent, Santa Clara,

California, USA). Only those samples with a 260 nm/280 nm ratio between 1.8–2.1, a 28S/18S ratio within 1.5–2, and RIN (>8) values qualified for a Poly-A enrichment strategy in order to generate the sequencing libraries applying the TruSeq mRNA Stranded Library Prep Kit (Illumina, Inc, California, USA). After Poly-A selection using Oligo-dT beads the mRNA was reverse-transcribed into cDNA. The cDNA was fragmented, end-repaired and poly-adenylated before ligation of TruSeq UD Indices (IDT, Coralville, Iowa, USA). The quality and quantity of the amplified sequencing libraries were validated using a Fragment Analyzer SS NGS Fragment Kit (1–6000 bp) (Agilent, Waldbronn, Germany). The equimolar pool of the samples was spiked into a NovaSeq6000 run targeting ~15M reads per sample on a S1 FlowCell (Novaseq S1 Reagent Kit, 100 cycles, Illumina, Inc, California, USA). Reads were quality-checked with FastQC. Sequencing adapters were removed with Trimmomatic[18] and aligned to the reference genome and transcriptome of Mus Musculus (GENCODE, GRCm38,p5) with STAR v2.7.3[19]. Distribution of the reads across genomic isoform expression was quantified using the R package GenomicRanges from Bioconductor Version 3.10. Minimum mapping quality, as well as minimum feature overlaps, was set to 10. Multi-overlaps were allowed. Differentially expressed genes (DEGs) were identified using the R package edgeR from Bioconductor Version 3.10, using a generalized linear model (glm) regression, a quasi-likelihood (QL) differential expression test and the trimmed means of M-values (TMM) normalization, thereby applying a FDR correction set at a 10% threshold ($q < 0.1$) and $p < 0.0012$. Only genes that passed this threshold were considered as significant DEGs.

Functional network prediction was generated through the use of QIAGEN's Ingenuity Pathway Analysis (IPA, QIAGEN Redwood City). IPA uses the curated Ingenuity Knowledge Base to identify the involvement of differentially expressed genes in specific diseases and cellular pathways, and to establish functional networks of direct and indirect interactions between differentially expressed genes based on a functional analysis algorithm[20]. For IPA, we only considered DEGs that passed the FDR-corrected threshold ($q < 0.1$ and $p < 0.0012$), as describe above.

Quantitative real-time PCR analysis

We used quantitative real-time PCR analyses (qRT-PCR) to verify the expression of the HERV-W ENV transcripts and to confirm differential expression of selected genes that were identified by the preceding RNAseq (see above). Isolation of whole hippocampi was performed as described above. For some experiments, whole hippocampi were further divided into dorsal and ventral parts. This was achieved by incisions at the dorsoventral midpoint, which was defined by the transition point where the hippocampal structure starts to curve more noticeably. RNA extraction were performed as described above. The resulting RNA was quantified by Nanodrop (DeNovix DS-11+ spectrophotometer, Labgene Scientific SA, Switzerland) and was reverse-transcribed into cDNA using iScript™ cDNA Synthesis Kit (Biorad, Hercules, California, USA) according to the manufacturer's instructions, after which it was stored at −80 °C until further use.

The Biorad CFX384TM Real-Time System (Biorad, Hercules, California, USA) using SYBR Green SsoAdvanced™ Universal SYBR® Green Supermix (Biorad, Hercules, California, USA) was used to measure the mRNA levels of the genes of interest. All primers were purchased from Eurofins Genomics GmbH (Germany). The samples were run in 384-well formats in triplicates as multiplexed reactions with a normalizing internal control (glyceraldehyde 3-phosphate dehydrogenase, GAPDH). The qRT-PCR reaction included 2.5 µl of 4ng/µl cDNA, 150 nM forward and reverse primers for each gene of interest, and 2.5 µl of SsoAdvanced™ Universal SYBR® Green Supermix (Biorad, Hercules, California, USA) in a total volume of 5 µl. Thermal cycling was initiated at 95 °C for 30 s, followed by 40 PCR cycles (10 s at 95 °C; 20 s at 60 °C), and a melt-curve analysis (65–95 °C, 0.5 °C increments at 5 sec/step). Each sample was analyzed in triplicate and a negative control (no template reaction) was included in each experiment to rule out any possible contamination. Relative target gene expression was calculated according to the $2^{-\Delta\Delta Ct}$ method.[21] Primer sequences of each gene of interest are summarized in **Supplementary Table 2**.

Gene	Forward primer sequence	Reverse primer sequence
Cacna1a	TTCCTCTACTATGCAGAATTCATTTTCT	CCGAGCCCGTACATTTTTATAAA
Cacna1g	TGCCTCTGAACACCAAGACTGTA	GCTCAGGTTGCCCTCATCAT
Gapdh	CCGGAAACCAGATCTCCCAC	CCCCCAGCTACAGAAAGGTC
HERV-W ENV	TTTACTCCTCTTTGGACCCT	ATCTGGGGTTCCATTTGAAG
Kmt2a	GTGACCTTGCCTAGTAATCGAACTT	CCGAATCGGACTAAACACTTTCC
Kmt2b	CGTCACCACCCTTGACTCC	CATGTAGCTGGGACAAGAGGA
Kmt2d	GTGGCTGTTCCACACCCAG	AGCTTGAGCTTCTCAGCATCG
Setd1a	TGGTCGAGAATGTGGCCTTT	TTCCTTGGCTTGCTGTTTGG
Shank3	GATCTGCCATCCCTACAAC	AGCTAAGGGTGAGCTAGGAT

Supplementary Table 2. List of forward and reverse primer sequences used in the qRT-PCR analyses of the target genes of interest. The list specifies forward and reverse primer sequences used for each target gene of interest, as well as for the reference housekeeping gene (*Gapdh*).

Western blot analysis

Protein extraction from hippocampal samples was conducted using the Thermo Scientific™ Subcellular Protein Fractionation Kit for Tissues (Thermo Fisher Scientific Inc., Massachusetts, USA, Prod.Nr: 87790) following the manufacturer's instructions. This extraction kit allows for the separation of cytoplasmic, membrane, nuclear soluble, and chromatin-bound protein extracts from tissue samples.

Chromatin-bound subcellular fractions hippocampal samples were prepared by taking 2.5µg of sample protein, 4x Laemmli buffer (Bio-Rad, catalogue number: 1610747) and 10% β-Mercaptoethanol. They were heated for 10 min at 96° and then loaded onto a GenScript SurePAGE™ Bis-Tris, 10 x 8, 4-20% gel (Genscript Biotech, Piscataway, USA, catalogue number: M00657). The gel was run for 3 hrs at 100mV in a Mini-PROTEAN Tetra Vertical Electrophoresis Cell (Bio-Rad, catalogue number: 1658004), with Tris-MOPS-SDS Running Buffer prepared according to manufacturer's instruction (Genscript, catalogue number: M00138). Afterwards, the proteins were transferred to an Immoblon-PSQ PVDF membrane (7 x 9 cm) (Merck, catalogue number: ISEQ00005). The Trans-Blot® Turbo™ Transfer System (BioRad) was used at 25V for 2 hrs using mPAGE Transfer Buffer (Genscript, catalogue number: MPTRB).

Following the transfer, the membrane was blocked in blocking buffer (PBS, 0.1% Tween 20, 5% skim milk powder) for 1 h at room temperature while shaking. The primary antibodies (see **Supplementary Table 3**) were diluted in blocking buffer according to manufacturer's instructions. The membrane was removed from the blocking buffer and incubated in the primary antibody solution overnight at 4°C with constant agitation. The next day, the membrane was washed 3 times for 10 min with PBS 0.1% Tween 20 while shaking. The membrane was incubated with the secondary antibody solution for 1h at room temperature while shaking, after which it was washed 3 times with PBS for 5 min each. After the last wash, SuperSignal™ West Pico PLUS Chemiluminescent Substrate (catalogue number: 34579) was added to the membranes to read the Chemiluminescence in a ChemiDoc XRS+ System (Bio-Rad) using the Image Lab™ Upgrade for ChemiDoc™ XRS+ System (Bio-Rad, catalogue number: 1708299). Tiff images were analyzed using the Fiji software. Levels of mono-, di-, and tri-methylation of H3K4 (H3K4me1, H3K4me2, and H3K4me3) and LSD1 were all analyzed with reference to H3 as the housekeeping control. All analyses were conducted by an experimenter who was blind to the genotype conditions. Blinding was performed in the form of coding by numbers.

**Target**	**Host**	**Conjugated**	**Company**	**Catalogue Nr.**
H3	Mouse	-	Abcam	10799
H3K4me1	Rabbit	-	Abcam	176877
H3K4me2	Rabbit	-	Abcam	32356
H3K4me3	Rabbit	-	Cell Signaling	9751S
IgG-mouse	Goat	HRP	Abcam	205719
IgG-rabbit	Donkey	HRP	GE Healthcare	NA934
LSD1	Rabbit	-	Cell Signaling	2139S

Supplementary Table 3. List of primary antibodies used for the Western blot analyses of the target proteins of interest.

Immunohistochemistry and microscopy

The density of excitatory (VGLUT1$^+$/PSD-95$^+$) and inhibitory (VGAT$^+$/Gephyrin$^+$) synapses and H3K4me2 protein levels were assessed in the hippocampus of WT and TG mice using immunohistochemistry and laser-scanning confocal microscopy. The animals were deeply anesthetized with an overdose of pentobarbital (Esconarkon ad us. vet., Streuli Pharma AG, Switzerland) and transcardially perfused with 20 ml of ice-cold PBS followed by 60 ml of ice-

cold 4% phosphate-buffered paraformaldehyde (PFA) with a perfusion rate of 20 ml/min. The brains were immediately removed from the skull and post-fixed in 4% PFA for 6 h before cryoprotection in 30% sucrose in PBS for 48 h. The brains were cut coronally with a sliding microtome at 30 μm (8 serial sections) and stored at − 20 °C in cryoprotectant solution (50 mM sodium phosphate buffer (pH 7.4) containing 15% glucose and 30% ethylene glycol; Sigma-Aldrich, Switzerland) until further processing.

Immunofluorescent stainings were performed according to previously established protocols.[7,16,22]. In brief, the slices were rinsed with Tris buffer (pH 7.4) before incubation with the primary antibody of interest (anti-Gephyrin: Synaptic Systems, catalogue number: 147011; dilution 1:1,000; anti-H3K4me2: Cell Signaling, catalogue number: 9725; dilution 1:1,000; anti-PSD-95: Thermo Fisher Scientific, catalogue number: 51–6900; anti-VGAT: Synaptic Systems, catalogue number: 131002; dilution 1:1,000; anti-VGLUT1: Synaptic Systems, catalogue number: 135304; dilution 1:1,000). The primary antibody was diluted in Tris buffer containing 0.2% Triton X-100 and 2% normal serum, and the sections were incubated free-floating under constant agitation (100 rpm) overnight at 4 °C. The sections were then washed three times for 10 min in Tris buffer, incubated for 30 min at room temperature with the secondary antibody coupled to either Alexa488 (Invitrogen, catalogue number: A21206; dilution 1:1,000) or Cy3 (Jackson ImmunoResearch Laboratories, catalogue number: 106–165-003; dilution 1:1,000). After incubation, which was shielded from light, the sections were washed thoroughly three times for 10 min in Tris buffer, mounted onto gelatinized glass slides, coverslipped with Dako fluorescence mounting medium and stored in the dark at 4 °C.

For H3K4me2, data collection was performed with confocal laser scanning microscopy (LSM-700; Zeiss, Jena, Germany) using a 20× (oil, NA 1.4) objective, as described before.[7,22] Laser intensities were kept constant during the entire image acquisition. For each animal, 5 images from 5 serial sections containing the hippocampal CA1, CA3 and dentate gyrus (DG) subregions (bregma: -1.6 to -2.8 mm) were acquired. H3K4me2 intensities (mean grey value) in each hippocampal subregion were averaged over the 5 images per animal. Quantification of H3K4me2 was achieved by means of NIH ImageJ software (https://imagej.nih.gov/ij/).

For co-localization studies of VGLUT1$^+$/PSD-95$^+$ (excitatory synapses) and VGAT$^+$/Gephyrin$^+$ (inhibitory synapses), double-immunofluorescent images of VGLUT1 and PSD-95, or VGAT and Gephyrin, were taken using sequential acquisition of separate wavelength channels by confocal laser scanning microscopy (Leica DMI6000 AFC, Model SP8, Mannheim, Germany) with a 63× (oil, NA 1.4) objective and a zoom of 1.8x. Laser intensities for each channel were set and kept constant during the entire image acquisition. For each animal, 3 single plane images were acquired for each subregion of the hippocampus (CA1, CA3 and DG), from 6 consecutive sections (bregma: -1.4 to -2.8 mm). Co-localization between VGLUT1$^+$ and PSD-95$^+$ puncta (excitatory synapses) or VGAT and Gephyrin (inhibitory synapses) were measured and calculated using a custom-made macro (kindly provided by Prof. emeritus Jean-Marc Fritschy, Institute of Pharmacology and Toxicology, University of Zurich, Switzerland) developed for the ImageJ software. This macro has been extensively validated for immunohistochemical co-localization studies and has been described in detail previously.[7] In brief, Gaussian filter, background subtraction and a threshold were applied to the images for each channel. The settings for each marker were adjusted so that an optimal representation of VGLUT1, PSD-95, VGAT and Gephyrin was achieved and kept constant during image analyses. The number of co-localized clusters was defined as pixel clusters in the presynaptic channel (VGLUT1 or VGAT) that overlapped with pixel clusters in the postsynaptic channel (PSD-95 or Gephyrin), with a set size cut-off at 0.05 μm^2.

All analyses were conducted by an experimenter who was blind to the genoytpe conditions. Blinding was performed in the form of coding by numbers.

Enzymatic activity of LSD1

The enzymatic activity of LSD1 was measured using the Epigenase™ LSD1 demethylase activity/inhibition assay (EpigenTek; Farmingdale, NY, USA; catalogue number: P-3079) following the manufacturer’s instruction. In brief, 5 µg of nuclear fractions from hippocampal samples were used. Well plates were prepared with sample triplicates, blank wells, and positive control wells. Assay buffer and substrate were added to these wells and incubated at 37°C for

105 minutes. Following this, the reaction solution was removed, and the wells were washed three times with 150 µl of wash buffer. Capture antibody solution was then added to all wells and incubated at room temperature for 60 minutes. After incubation, the capture antibody solution was removed, and the wells were washed three times with 150 µl of wash buffer. Subsequently, detection antibody solution was added and incubated for 30 minutes. After incubation, the wells were washed four times with 150 µl of wash buffer. Finally, fluorescence development solution was added to each well for 2-4 minutes, after which the fluorescence was read using a fluorescence microplate reader (Ex/Em = 530/590 nm).

Statistical analyses

All statistical analyses of behavioral, cognitive, Western blot, RT-qPCR, and immunohistochemical data were performed using Statistical Package for the Social Sciences (SPSS) Statistics (version 29.0, IBM, Armonk, NY, USA) and Prism (version 10.0; GraphPad Software, La Jolla, California), with statistical significance set at $p < 0.05$ unless specified otherwise. All behavioral, cognitive, Western blot, RT-qPCR, and immunohistochemical data met the assumptions of normal distribution and equality of variance. F-tests for equality of variances were used to ascertain the variance of data between WT and TG mice. Given the exploratory nature of our study, no sample size estimate was calculated to detect a pre-specified effect. Exclusion of animals was not applied.

All basal Western blot, RT-qPCR, and immunohistochemical data were analyzed using independent Student's *t* tests (two-tailed). Western blot data that were generated after VEH or ORY-1001 treatment were analyzed using 2 × 2 (genotype × treatment) analysis of variance (ANOVA), followed by Tukey's post-hoc test for multiple comparisons whenever appropriate. All behavioral data generated during the open field test and marble burying test, which did not involve additional drug treatments, were also were analyzed using independent Student's *t* tests (two-tailed). All open field and marble burying data that were generated after VEH or ORY-1001 treatment were analyzed using 2 × 2 (genotype × treatment) analysis of variance (ANOVA), followed by Tukey's post-hoc test for multiple comparisons whenever appropriate. Absolute

exploration times in the social interaction and novel object recognition test were analyzed using 2 × 2 (genotype × object) repeated-measures ANOVA (RM-ANOVA), followed by Šidák post-hoc tests for multiple comparisons whenever appropriate. Social preference, social memory, and novel object recognition indices were analyzed using independent Student's *t* tests (two-tailed). After VEH or ORY-1001 treatment, absolute exploration times in the social interaction and novel object recognition test were analyzed using 2 × 2 × 2 (genotype × object × treatment) RM-ANOVA, followed by Šidák post-hoc tests for multiple comparisons whenever appropriate. Social preference, social memory, and novel object recognition indices in VEH- or ORY-1001-treated animals were analyzed using 2 × 2 (genotype × treatment) ANOVA, followed by Tukey's post-hoc test for multiple comparisons whenever appropriate. Basal % PPI scores and acoustic startle reactivity were analyzed using 2 × 3 × 3 (genotype × prepulse × pulse) and 2 × 3 (genotype × pulse) RM-ANOVAs, respectively. After VEH or ORY-1001 treatment, these variables were analyzed using 2 × 3 × 3 × 2 (genotype × prepulse × pulse × treatment) and 2 × 3 × 2 (genotype × pulse × treatment) RM-ANOVAs, followed by Šidák post-hoc tests for multiple comparisons whenever appropriate.

Transcriptomic data were analyzed as described above, using FDR correction set at a 10% threshold ($q < 0.1$) and $p < 0.0012$.

Supplementary Data

Supplementary Figures

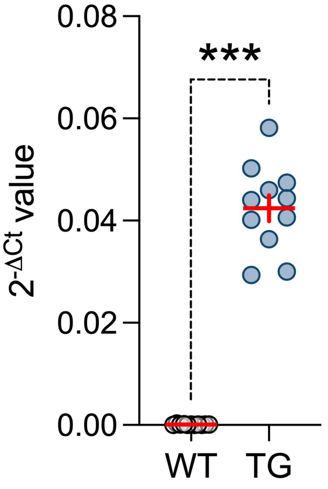

Supplementary Figure S1. Confirmation of HERV-W ENV expression in adult hippocampal tissue. HERV-W ENV mRNA levels were quantified in male transgenic CAG$^{HERV\text{-}Wenv}$ mice (TG) and wild-type (WT) littermates using quantitative real-time PCR. Note the absence of HERV-W ENV transcripts in WT mice. ***$p < 0.001$ ($t_{(20)} = 16.54$; 2-tailed); $n = 11$ per genotype. Consistent results were obtained in analyses of other brain regions (not shown).

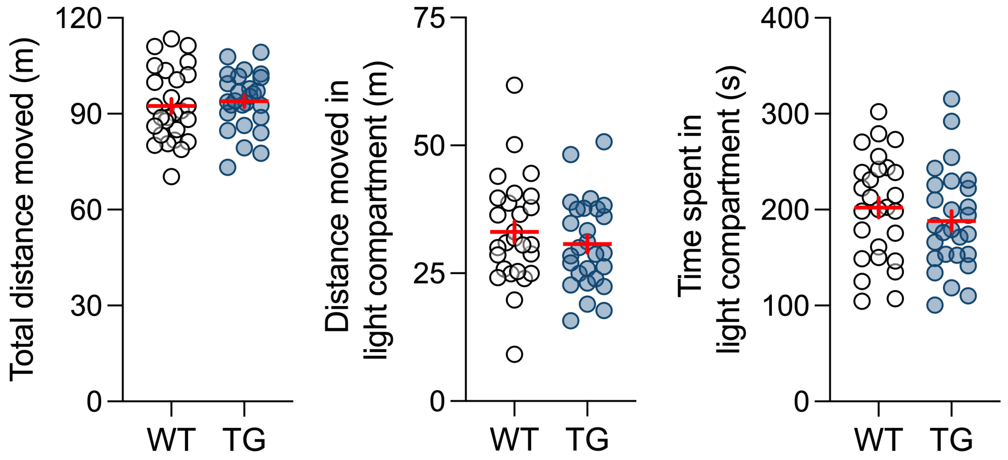

Supplementary Figure S2. Total distance moved, distance moved in the light compartment, and time spent in the light compartment from adult male transgenic CAG$^{HERV\text{-}Wenv}$ mice (TG) and wild-type (WT) littermates during the light-dark box test of innate anxiety-like behavior. All scatter plots show individual mice with overlaid group means ± s.e.m. and are based on $n = 27$ mice per each genotype.

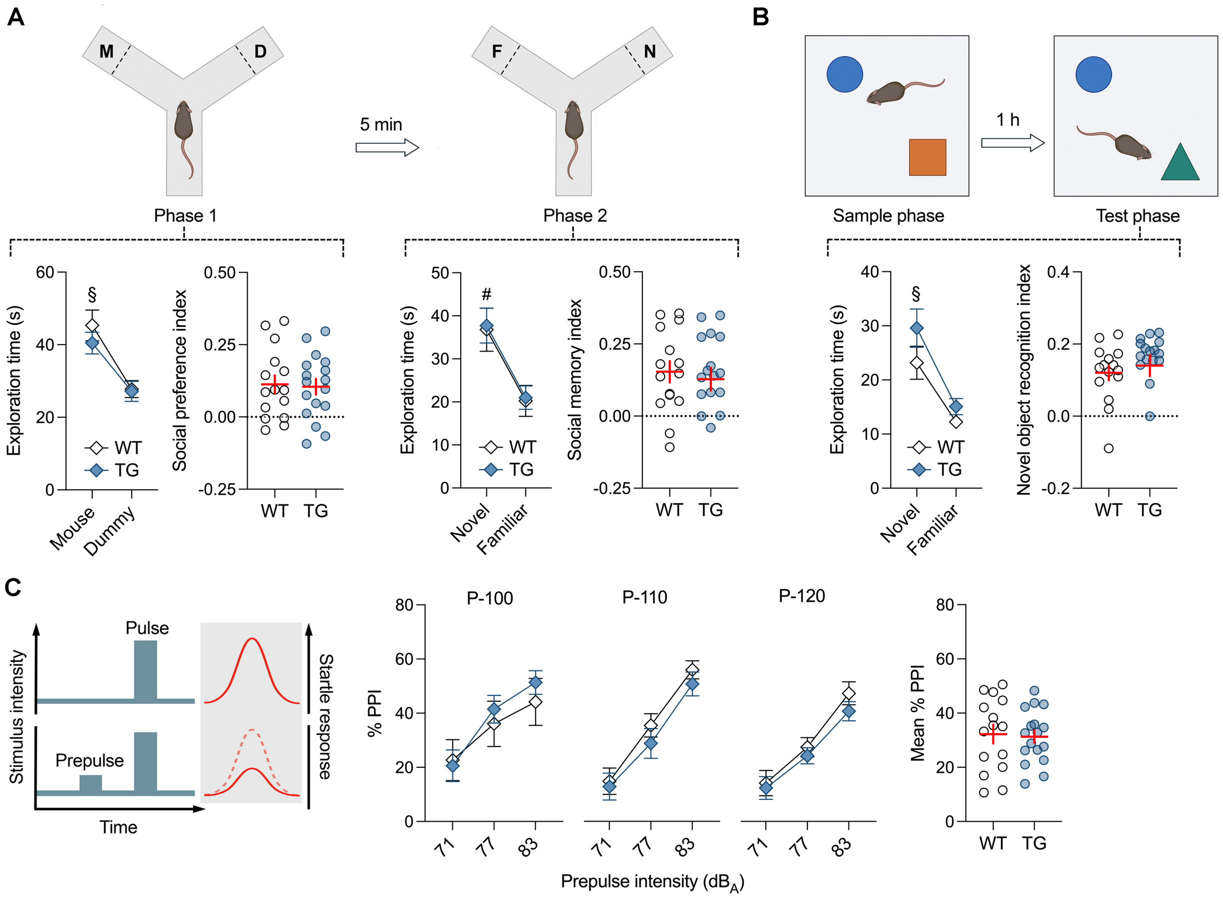

Supplementary Figure S3. Behavioral and cognitive phenotypes of adolescent mice expressing HERV-W ENV. All data were obtained from male transgenic CAG$^{HERV\text{-}Wenv}$ mice (TG) and wild-type (WT) littermates when they reached 4 to 6 weeks of age. **(A)** Phase 1 (D = dummy object, M = unfamiliar mouse) and phase 2 (F = familiar mouse; N = novel mouse) of the social interaction test, which assessed sociability and social memory, respectively. The line plots show the time spent with the unfamiliar mouse or dummy object in phase 1 of the test, or with the novel or familiar mouse in phase 2 of the test. The scatter plots depict the social preference index (values > 0 represent a preference toward the unfamiliar mouse) in phase 1 and the social memory index (values > 0 represent a preference toward the novel mouse) in phase 2 of the test. $^{\S}p < 0.001$ ($F_{(1,30)} = 20.23$) and $^{\#}p < 0.001$ ($F_{(1,30)} = 25.82$), reflecting the significant main effects of object in repeated-measures ANOVA of exploration time in phase 1 and 2, respectively. **(B)** Object recognition memory in the novel object recognition test, in which the animals were required to discriminate a novel object from a previously familiarized object. The line plot shows the time spent with the novel or familiar object during the test phase, whereas the scatter plot depicts the novel object recognition index (values > 0 represent a preference toward the novel object). $^{\S}p < 0.001$ ($F_{(1,30)} = 39.34$), reflecting the significant main effect of object in repeated-measures ANOVA of exploration time. **(C)** Prepulse inhibition (PPI) test of sensorimotor gating using 3 prepulse intensities (71, 77 and 83 dB_A) and 3 pulse intensities (P-100, P-110 and P-120, which correspond to 100, 110 and 120 dBA). The line plots show % PPI as a function of prepulse and pulse

intensities, whereas the scatter plot depicts the mean % PPI across all prepulse and pulse intensities. All scatter plots show individual mice with overlaid group means ± s.e.m; all line plots show group means ± s.e.m. All data are based on *n*(WT) = 15 and *n*(TG) = 17 mice per test.

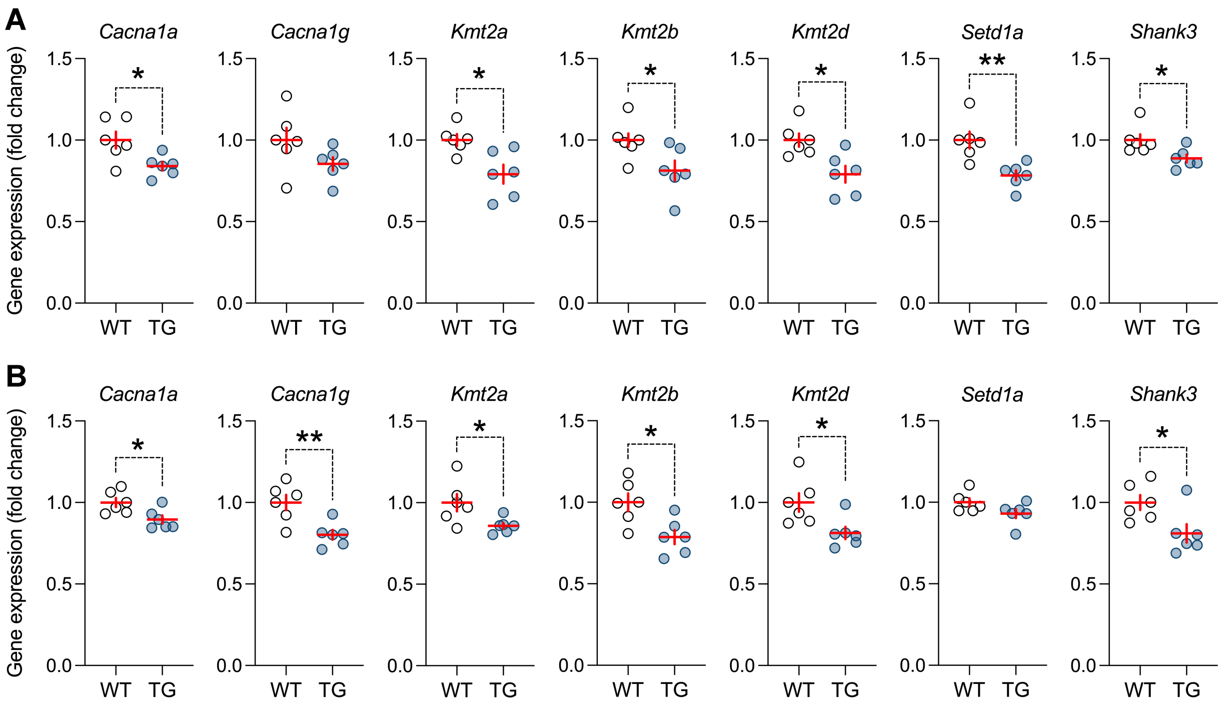

Supplementary Figure S4. Gene expression analysis of selected candiate genes in the dorsal and ventral hippocampus of transgenic $CAG^{HERV\text{-}Wenv}$ mice (TG) relative to wild-type (WT) littermates, as assessed using qRT-PCR. **(A)** Fold changes of selected genes in the dorsal hippocampus. *$p < 0.05$ and **$p < 0.01$, based on independent *t*-tests (*Cacna1a*: $t_{(10)} = 2.74$; *Kmt2a*: $t_{(10)} = 3.05$; *Kmt2b*: $t_{(10)} = 2.56$; *Kmt2d*: $t_{(10)} = 3.16$; *Setd1a*: $t_{(10)} = 3.62$; *Shank3*: $t_{(10)} = 2.63$). **(B)** Fold changes of selected genes in the ventral hippocampus. *$p < 0.05$ and **$p < 0.01$, based on independent *t*-tests (*Cacna1a*: $t_{(10)} = 2.79$; *Cacna1g*: $t_{(10)} = 3.52$; *Kmt2a*: $t_{(10)} = 2.52$; *Kmt2b*: $t_{(10)} = 3.06$; *Kmt2d*: $t_{(10)} = 2.72$; *Shank3*: $t_{(10)} = 2.61$). All scatter plots show individual mice with overlaid group means ± s.e.m.; *n* = 6 per genotype.

Supplementary Tables

Supplementary Table 4. Summary of differential gene expression in the prefrontal cortex of male transgenic $CAG^{HERV\text{-}Wenv}$ mice (TG; *n* = 3) relative to wild-type (WT; *n* = 3) littermates, as assessed using next-generation RNA sequencing. The table lists all genes that were differentially expressed in TG relative to WT mice based on a false-discovery rate (FDR) correction set at a 10% threshold ($q < 0.1$) and $p < 0.0012$. The table specifies the gene symbols, gene IDs, magnitude of gene expression changes (log2 fold change, Log2FC), *q*-values and *p*-values. The genes are sorted according to *q*-values.

Gene symbol	Gene ID	Log2 FC	*q*-value	*p*-value
**Zbtb16**	ENSMUSG00000070304	-0.601	2.98E-06	3.62E-10
**Rnf26rt**	ENSMUSG00000027273	-7.345	2.22E-05	4.04E-09
**Scn2b**	ENSMUSG00000030270	0.345	8.33E-05	2.02E-08
**Fxyd2**	ENSMUSG00000036264	-0.962	7.43E-04	2.25E-07
**Cd99l2**	ENSMUSG00000027438	0.292	6.04E-03	2.20E-06

Supplementary Table 5. Summary of differential gene expression in the hippocampus of male transgenic $CAG^{HERV\text{-}Wenv}$ mice (TG; *n* = 3) relative to wild-type (WT; *n* = 3) littermates, as assessed using next-generation RNA sequencing. The table lists all genes that were differentially expressed in TG relative to WT mice based on a false-discovery rate (FDR) correction set at a 10% threshold ($q < 0.1$) and $p < 0.0012$. The table specifies the gene symbols, gene IDs, magnitude of gene expression changes (log2 fold change, Log2FC), *q*-values and *p*-values. The genes are sorted according to *q*-values.

Gene symbol	Gene ID	Log2 FC	*q*-value	*p*-value
**Scn2b**	ENSMUSG00000066687	0.336	3.25E-05	7.80E-09
**Snap25**	ENSMUSG00000072476	0.442	1.23E-04	3.70E-08
**Cpne9**	ENSMUSG00000070304	-0.788	4.25E-04	1.53E-07
**Fstl4**	ENSMUSG00000059412	-0.765	5.28E-04	2.22E-07
**Napb**	ENSMUSG00000027438	0.360	1.38E-03	6.64E-07
**Cacybp**	ENSMUSG00000014226	0.302	1.98E-03	1.16E-06
**Tmem267**	ENSMUSG00000074634	0.900	1.98E-03	1.19E-06
**Cacna1a**	ENSMUSG00000034656	-0.349	3.99E-03	2.64E-06
**Hspa8**	ENSMUSG00000015656	0.422	6.08E-03	4.38E-06
**Extl3**	ENSMUSG00000021978	-0.309	6.10E-03	4.76E-06
**Rims3**	ENSMUSG00000032890	-0.931	6.57E-03	5.53E-06
**Plec**	ENSMUSG00000022565	-0.390	8.52E-03	7.81E-06
**Htr3a**	ENSMUSG00000032269	0.789	8.52E-03	8.38E-06
**Zbtb16**	ENSMUSG00000066687	-0.635	8.52E-03	9.14E-06

**Srcap**	ENSMUSG00000053877	-0.416	8.52E-03	9.41E-06
**Tnrc18**	ENSMUSG00000039477	-0.489	8.52E-03	9.72E-06
**Gm42770**	ENSMUSG00000105983	-0.903	8.99E-03	1.13E-05
**Huwe1**	ENSMUSG00000025261	-0.215	8.99E-03	1.14E-05
**Kcnq3**	ENSMUSG00000056258	-0.317	8.99E-03	1.19E-05
**Elovl6**	ENSMUSG00000041220	0.285	9.50E-03	1.32E-05
**Pkd1**	ENSMUSG00000032855	-0.341	9.50E-03	1.37E-05
**Kcnc3**	ENSMUSG00000062785	-0.535	1.55E-02	2.33E-05
**Gm42772**	ENSMUSG00000105700	-0.883	1.58E-02	2.47E-05
**Map1a**	ENSMUSG00000027254	-0.408	1.67E-02	2.70E-05
**Nav2**	ENSMUSG00000052512	-0.522	1.70E-02	2.86E-05
**Ptchd4**	ENSMUSG00000042256	-0.751	1.82E-02	3.17E-05
**Mast4**	ENSMUSG00000034751	-0.361	2.01E-02	3.80E-05
**Agpat4**	ENSMUSG00000023827	0.354	2.01E-02	3.84E-05
**Srrm4**	ENSMUSG00000063919	-0.680	2.01E-02	3.87E-05
**Swi5**	ENSMUSG00000044627	0.222	2.01E-02	3.97E-05
**Gm43320**	ENSMUSG00000106414	-0.973	2.09E-02	4.37E-05
**Foxk1**	ENSMUSG00000056493	-0.468	2.09E-02	4.51E-05
**Camta2**	ENSMUSG00000040712	-0.320	2.16E-02	4.86E-05
**Szt2**	ENSMUSG00000033253	-0.389	2.16E-02	4.92E-05
**Dagla**	ENSMUSG00000035735	-0.441	2.33E-02	5.45E-05
**Krba1**	ENSMUSG00000042810	-0.342	2.36E-02	5.67E-05
**Glra2**	ENSMUSG00000018589	0.389	2.43E-02	5.99E-05
**Megf8**	ENSMUSG00000045039	-0.249	2.43E-02	6.12E-05
**Ccl28**	ENSMUSG00000074715	1.148	2.46E-02	6.34E-05
**Nlrx1**	ENSMUSG00000032109	0.589	2.55E-02	6.84E-05
**Kmt2d**	ENSMUSG00000048154	-0.614	2.55E-02	7.03E-05
**Ankrd52**	ENSMUSG00000014498	-0.399	2.55E-02	7.05E-05
**Dnaja1**	ENSMUSG00000028410	0.198	3.03E-02	8.56E-05
**Ptprm**	ENSMUSG00000033278	-0.373	3.19E-02	9.18E-05
**Zwint**	ENSMUSG00000019923	0.214	3.29E-02	9.76E-05
**Lrp1**	ENSMUSG00000040249	-0.301	3.29E-02	9.88E-05
**Ccdc25**	ENSMUSG00000022035	0.329	3.36E-02	1.04E-04
**Gm43319**	ENSMUSG00000105958	-0.938	3.36E-02	1.06E-04
**Gm42715**	ENSMUSG00000107023	-0.408	3.36E-02	1.07E-04
**Apc2**	ENSMUSG00000020135	-0.318	3.50E-02	1.15E-04
**Gm48261**	ENSMUSG00000113073	-1.308	3.50E-02	1.16E-04
**Gm42992**	ENSMUSG00000106320	-0.954	3.78E-02	1.27E-04
**Nfic**	ENSMUSG00000055053	-0.443	3.86E-02	1.32E-04
**Rnf26**	ENSMUSG00000053128	0.422	3.86E-02	1.34E-04
**Mrps12**	ENSMUSG00000045948	0.274	3.86E-02	1.37E-04
**Kmt2b**	ENSMUSG00000006307	-0.424	3.87E-02	1.42E-04

**Crebbp**	ENSMUSG00000022521	-0.351	3.87E-02	1.43E-04
**Cic**	ENSMUSG00000005442	-0.424	3.87E-02	1.44E-04
**D10Wsu102e**	ENSMUSG00000020255	-0.436	3.89E-02	1.49E-04
**2900064K03Rik**	ENSMUSG00000105771	-0.887	3.89E-02	1.52E-04
**Snx10**	ENSMUSG00000038301	0.234	3.89E-02	1.52E-04
**Hsp90aa1**	ENSMUSG00000021270	0.354	3.89E-02	1.54E-04
**Wnk2**	ENSMUSG00000037989	-0.562	3.96E-02	1.59E-04
**Nf1**	ENSMUSG00000020716	-0.211	4.02E-02	1.64E-04
**Rwdd2a**	ENSMUSG00000032417	0.363	4.21E-02	1.77E-04
**Hcn2**	ENSMUSG00000020331	-0.354	4.21E-02	1.79E-04
**Ank3**	ENSMUSG00000069601	-0.300	4.21E-02	1.81E-04
**Morf4l2**	ENSMUSG00000031422	0.204	4.21E-02	1.82E-04
**Rgs5**	ENSMUSG00000026678	0.241	4.28E-02	1.90E-04
**Bsn**	ENSMUSG00000032589	-0.420	4.28E-02	1.92E-04
**Hmgb2**	ENSMUSG00000054717	0.925	4.28E-02	1.94E-04
**Gm43317**	ENSMUSG00000104655	-0.790	4.28E-02	1.95E-04
**Dscam**	ENSMUSG00000050272	-0.365	4.37E-02	2.02E-04
**Cblb**	ENSMUSG00000022637	-0.478	4.49E-02	2.10E-04
**AI593442**	ENSMUSG00000078307	-0.539	4.80E-02	2.35E-04
**Ppia**	ENSMUSG00000071866	0.196	4.80E-02	2.36E-04
**Syt14**	ENSMUSG00000016200	-0.335	4.80E-02	2.38E-04
**Rorb**	ENSMUSG00000036192	-0.515	4.80E-02	2.42E-04
**Gm42993**	ENSMUSG00000105985	-0.947	4.80E-02	2.42E-04
**Hsbp1**	ENSMUSG00000031839	0.214	4.80E-02	2.45E-04
**Rai1**	ENSMUSG00000062115	-0.326	4.80E-02	2.47E-04
**Pdzd2**	ENSMUSG00000022197	-0.434	4.80E-02	2.48E-04
**Kmt2a**	ENSMUSG00000002028	-0.434	4.80E-02	2.52E-04
**Calr**	ENSMUSG00000003814	0.245	4.80E-02	2.54E-04
**Tmem131**	ENSMUSG00000026116	-0.410	4.80E-02	2.57E-04
**Psma4**	ENSMUSG00000032301	0.254	4.91E-02	2.66E-04
**Dot1l**	ENSMUSG00000061589	-0.583	4.96E-02	2.72E-04
**Plxdc1**	ENSMUSG00000017417	-0.876	4.96E-02	2.74E-04
**Sik3**	ENSMUSG00000034135	-0.328	5.03E-02	2.81E-04
**Tpi1**	ENSMUSG00000023456	0.207	5.09E-02	2.87E-04
**Rskr**	ENSMUSG00000037593	-0.818	5.15E-02	2.96E-04
**Gm42771**	ENSMUSG00000106579	-0.832	5.15E-02	2.97E-04
**Ubr4**	ENSMUSG00000066036	-0.289	5.17E-02	3.01E-04
**Pde1a**	ENSMUSG00000059173	0.546	5.33E-02	3.13E-04
**Gm47087**	ENSMUSG00000111212	-0.897	5.43E-02	3.26E-04
**Syvn1**	ENSMUSG00000024807	-0.425	5.43E-02	3.26E-04
**Setd1a**	ENSMUSG00000042308	-0.364	5.46E-02	3.31E-04
**Tmem50b**	ENSMUSG00000022964	0.234	5.46E-02	3.35E-04

**Srrm2**	ENSMUSG00000039218	-0.361	5.46E-02	3.38E-04
**Canx**	ENSMUSG00000020368	0.187	5.46E-02	3.41E-04
**Ep400**	ENSMUSG00000029505	-0.310	5.62E-02	3.54E-04
**Tpsb2**	ENSMUSG00000033825	4.446	5.62E-02	3.57E-04
**Gpx4**	ENSMUSG00000075706	0.285	5.62E-02	3.61E-04
**Ankrd34c**	ENSMUSG00000047606	-0.903	5.62E-02	3.64E-04
**Gdi1**	ENSMUSG00000015291	0.173	5.67E-02	3.71E-04
**Ttr**	ENSMUSG00000061808	8.335	6.04E-02	3.99E-04
**Ptgs2**	ENSMUSG00000032487	0.485	6.04E-02	4.02E-04
**Dynll1**	ENSMUSG00000009013	0.259	6.07E-02	4.08E-04
**Mea1**	ENSMUSG00000002768	0.220	6.24E-02	4.23E-04
**Caln1**	ENSMUSG00000060371	0.343	6.35E-02	4.35E-04
**Pttg1**	ENSMUSG00000020415	0.938	6.37E-02	4.40E-04
**Ociad2**	ENSMUSG00000029153	0.363	6.70E-02	4.72E-04
**Atp5h**	ENSMUSG00000034566	0.187	6.70E-02	4.81E-04
**Rsph4a**	ENSMUSG00000039552	1.680	6.70E-02	4.82E-04
**Vars**	ENSMUSG00000007029	-0.329	6.70E-02	4.85E-04
**Ncor2**	ENSMUSG00000029478	-0.479	6.70E-02	4.86E-04
**Scrt1**	ENSMUSG00000048385	-0.483	6.70E-02	4.87E-04
**Nr1d1**	ENSMUSG00000020889	-0.621	6.88E-02	5.04E-04
**Htt**	ENSMUSG00000029104	-0.245	7.00E-02	5.21E-04
**Dennd4b**	ENSMUSG00000042404	-0.365	7.10E-02	5.37E-04
**Gm42995**	ENSMUSG00000105495	-1.203	7.10E-02	5.38E-04
**Hectd4**	ENSMUSG00000042744	-0.343	7.10E-02	5.41E-04
**Col23a1**	ENSMUSG00000063564	1.047	7.13E-02	5.56E-04
**1700001L19Rik**	ENSMUSG00000021534	0.650	7.13E-02	5.56E-04
**Prps1**	ENSMUSG00000031432	0.285	7.13E-02	5.56E-04
**Gm37060**	ENSMUSG00000103808	-0.834	7.22E-02	5.68E-04
**Arid1b**	ENSMUSG00000069729	-0.211	7.25E-02	5.74E-04
**Fgf11**	ENSMUSG00000042826	0.417	7.32E-02	5.91E-04
**Mrtfa**	ENSMUSG00000042292	-0.293	7.32E-02	5.94E-04
**Rad23b**	ENSMUSG00000028426	0.268	7.32E-02	5.96E-04
**Ltbp4**	ENSMUSG00000040488	-0.472	7.32E-02	6.01E-04
**Khsrp**	ENSMUSG00000007670	-0.287	7.32E-02	6.02E-04
**Nxpe4**	ENSMUSG00000044229	0.365	7.39E-02	6.16E-04
**Syn3**	ENSMUSG00000059602	-0.779	7.39E-02	6.16E-04
**Gm50403**	ENSMUSG00000117943	-0.793	7.44E-02	6.27E-04
**Kmt2c**	ENSMUSG00000038056	-0.243	7.44E-02	6.29E-04
**Sptb**	ENSMUSG00000021061	-0.298	7.46E-02	6.36E-04
**Shank1**	ENSMUSG00000038738	-0.595	7.46E-02	6.44E-04
**Usp2**	ENSMUSG00000032010	-0.299	7.46E-02	6.45E-04
**Wdfy3**	ENSMUSG00000043940	-0.257	7.56E-02	6.60E-04

**Auts2**	ENSMUSG00000029673	-0.720	7.56E-02	6.62E-04
**Slc25a37**	ENSMUSG00000034248	-0.389	7.57E-02	6.73E-04
**Ccdc181**	ENSMUSG00000026578	0.360	7.57E-02	6.81E-04
**Ralgapa2**	ENSMUSG00000037110	-0.328	7.57E-02	6.82E-04
**Zdhhc8**	ENSMUSG00000060166	-0.379	7.57E-02	6.85E-04
**Padi6**	ENSMUSG00000040935	2.368	7.57E-02	6.86E-04
**Gm45461**	ENSMUSG00000109744	-0.842	7.62E-02	6.95E-04
**Sytl5**	ENSMUSG00000054453	0.678	7.68E-02	7.10E-04
**Agap1**	ENSMUSG00000055013	-0.258	7.68E-02	7.12E-04
**Map1s**	ENSMUSG00000019261	-0.309	7.68E-02	7.14E-04
**Stard8**	ENSMUSG00000031216	-0.352	8.04E-02	7.53E-04
**Bcl9l**	ENSMUSG00000063382	-0.459	8.04E-02	7.57E-04
**Fasn**	ENSMUSG00000025153	-0.239	8.06E-02	7.64E-04
**Ttc28**	ENSMUSG00000033209	-0.495	8.09E-02	7.72E-04
**Arpc5**	ENSMUSG00000008475	0.236	8.14E-02	7.84E-04
**Il1rap**	ENSMUSG00000022514	-0.386	8.14E-02	7.87E-04
**Shank3**	ENSMUSG00000022623	-0.439	8.17E-02	7.98E-04
**Ubn2**	ENSMUSG00000038538	-0.273	8.17E-02	8.02E-04
**Il1rapl1**	ENSMUSG00000052372	-0.377	8.17E-02	8.05E-04
**Celsr2**	ENSMUSG00000068740	-0.352	8.25E-02	8.17E-04
**Prrc2a**	ENSMUSG00000024393	-0.405	8.25E-02	8.22E-04
**Mcfd2**	ENSMUSG00000024150	0.228	8.25E-02	8.27E-04
**Crtc1**	ENSMUSG00000003575	-0.439	8.27E-02	8.34E-04
**Hlf**	ENSMUSG00000003949	-0.589	8.65E-02	8.77E-04
**Pcnp**	ENSMUSG00000071533	0.246	8.80E-02	9.07E-04
**Arid1a**	ENSMUSG00000007880	-0.399	8.80E-02	9.11E-04
**Bmp1**	ENSMUSG00000022098	-0.478	8.80E-02	9.19E-04
**Abcc8**	ENSMUSG00000040136	-0.498	8.80E-02	9.20E-04
**Mcpt4**	ENSMUSG00000061068	3.877	8.80E-02	9.20E-04
**Pde7b**	ENSMUSG00000019990	-0.643	8.97E-02	9.42E-04
**Zfp39**	ENSMUSG00000037001	0.317	9.10E-02	9.71E-04
**Slc5a5**	ENSMUSG00000000792	0.501	9.10E-02	9.74E-04
**Zswim8**	ENSMUSG00000021819	-0.252	9.10E-02	9.86E-04
**Gm47590**	ENSMUSG00000112601	-0.866	9.10E-02	9.94E-04
**Gabrd**	ENSMUSG00000029054	-0.600	9.10E-02	9.97E-04
**Gm48582**	ENSMUSG00000114501	-1.040	9.10E-02	9.97E-04
**Rab8b**	ENSMUSG00000036943	0.342	9.10E-02	9.97E-04
**Cox7c**	ENSMUSG00000017778	0.202	9.10E-02	9.99E-04
**D630023F18Rik**	ENSMUSG00000044816	0.552	9.26E-02	1.02E-03
**Sipa1l3**	ENSMUSG00000030583	-0.374	9.30E-02	1.03E-03
**Gm42991**	ENSMUSG00000106386	-0.963	9.30E-02	1.04E-03
**Prss35**	ENSMUSG00000033491	0.844	9.43E-02	1.06E-03

**Jmjd1c**	ENSMUSG00000037876	-0.201	9.43E-02	1.06E-03
**Raph1**	ENSMUSG00000026014	-0.322	9.44E-02	1.07E-03
**Praf2**	ENSMUSG00000031149	0.211	9.44E-02	1.08E-03
**Fus**	ENSMUSG00000030795	-0.496	9.45E-02	1.08E-03
**Bcar1**	ENSMUSG00000031955	-0.292	9.45E-02	1.09E-03
**Slc9a2**	ENSMUSG00000026062	0.402	9.68E-02	1.12E-03
**Gm49380**	ENSMUSG00000111409	0.419	9.74E-02	1.14E-03
**Sbf1**	ENSMUSG00000036529	-0.231	9.74E-02	1.14E-03
**Cacna1g**	ENSMUSG00000020866	-0.738	9.81E-02	1.15E-03
**Atg2a**	ENSMUSG00000024773	-0.307	9.84E-02	1.16E-03
**Pitpnc1**	ENSMUSG00000040430	-0.471	9.87E-02	1.17E-03
**Scaf1**	ENSMUSG00000038406	-0.278	9.94E-02	1.19E-03

SUPPLEMENTARY REFERENCES

1. Belzung C, Griebel G. Measuring normal and pathological anxiety-like behaviour in mice: a review. Behav Brain Res. 2001;125:141-149.
2. Thomas A, Burant A, Bui N, Graham D, Yuva-Paylor LA, Paylor R. Marble burying reflects a repetitive and perseverative behavior more than novelty-induced anxiety. Psychopharmacology. 2009;204:361-373.
3. Angoa-Pérez M, Kane MJ, Briggs DI, Francescutti DM, Kuhn DM. Marble burying and nestlet shredding as tests of repetitive, compulsive-like behaviors in mice. J Vis Exp. 2013;(82):50978.
4. Sungur AÖ, Vörckel KJ, Schwarting RK, Wöhr M. Repetitive behaviors in the Shank1 knockout mouse model for autism spectrum disorder: developmental aspects and effects of social context. J Neurosci Methods. 2014;234:92-100.
5. Dhamne SC, Silverman JL, Super CE, Lammers SHT, Hameed MQ, Modi ME, et al. Replicable in vivo physiological and behavioral phenotypes of the Shank3B null mutant mouse model of autism. Mol Autism. 2017;8:26.
6. Vuillermot S, Luan W, Meyer U, Eyles D. Vitamin D treatment during pregnancy prevents autism-related phenotypes in a mouse model of maternal immune activation. Mol Autism. 2017;8:9.
7. Schalbetter SM, von Arx AS, Cruz-Ochoa N, Dawson K, Ivanov A, Mueller FS, et al. Adolescence is a sensitive period for prefrontal microglia to act on cognitive development. Sci Adv. 2022; 8:eabi6672.
8. Cohen SJ, Munchow AH, Rios LM, Zhang G, Asgeirsdóttir HN, Stackman RW Jr. The rodent hippocampus is essential for nonspatial object memory. Curr Biol. 2013;23:1685-90.
9. Chao OY, Nikolaus S, Yang YM, Huston JP. Neuronal circuitry for recognition memory of object and place in rodent models. Neurosci Biobehav Rev. 2022;141:104855.
10. Swerdlow NR, Weber M, Qu Y, Light GA, Braff DL. Realistic expectations of prepulse inhibition in translational models for schizophrenia research. Psychopharmacology 2008; 199(3):331-388.
11. Swerdlow NR, Light GA, Thomas ML, Sprock J, Calkins ME, Green MF, et al. Deficient prepulse inhibition in schizophrenia in a multi-site cohort: Internal replication and extension. Schizophr Res. 2018;198:6-15.
12. Herrero F, Mueller FS, Gruchot J, Küry P, Weber-Stadlbauer U, Meyer U. Susceptibility and resilience to maternal immune activation are associated with differential expression of endogenous retroviral elements. Brain Behav Immun. 2023; 107:201-214.
13. Meyer U, Feldon J, Schedlowski M, Yee BK. Towards an immuno-precipitated neurodevelopmental animal model of schizophrenia. Neurosci Biobehav Rev. 2005;29:913-947.

14. Weber-Stadlbauer U, Richetto J, Labouesse MA, Bohacek J, Mansuy IM, Meyer U. Transgenerational transmission and modification of pathological traits induced by prenatal immune activation. Mol Psychiatry. 2017; 22:102-112.
15. Scarborough J, Mueller F, Arban R, Dorner-Ciossek C, Weber-Stadlbauer U, Rosenbrock H, et al. Preclinical validation of the micropipette-guided drug administration (MDA) method in the maternal immune activation model of neurodevelopmental disorders. Brain Behav Immun. 2020; 88:461-470.
16. Schalbetter SM, Mueller FS, Scarborough J, Richetto J, Weber-Stadlbauer U, Meyer U, et al. Oral application of clozapine-N-oxide using the micropipette-guided drug administration (MDA) method in mouse DREADD systems. Lab Anim. 2021; 50:69-75.
17. Mattei D, Ivanov A, van Oostrum M, Pantelyushin S, Richetto J, Mueller F, et al. Enzymatic dissociation induces transcriptional and proteotype bias in brain cell populations. Int J Mol Sci. 2020;21:7944.
18. Bolger AM, Lohse M, Usadel B. Trimmomatic: a flexible trimmer for Illumina sequence data. Bioinformatics. 2014; 30:2114-2120.
19. Dobin A, Davis CA, Schlesinger F, Drenkow J, Zaleski C, Jha S et al. STAR: ultrafast universal RNA-seq aligner. Bioinformatics. 2013;29:15-21.
20. Thomas S, Bonchev D. A survey of current software for network analysis in molecular biology. Hum Genomics. 2010;4:353-360.
21. Livak KJ, Schmittgen TD. Analysis of relative gene expression data using real-time quantitative PCR and the 2(-Delta Delta C(T)) Method. Methods. 2001;25:402-408.
22. Notter T, Schalbetter SM, Clifton NE, Mattei D, Richetto J, Thomas K, et al. Neuronal activity increases translocator protein (TSPO) levels. Mol Psychiatry. 2021;26:2025-2037.
